# p-CuO/n-ZnO Heterojunction Pyro-Phototronic Photodetector Controlled by CuO Preparation Parameters

**DOI:** 10.3390/s24248197

**Published:** 2024-12-22

**Authors:** Zhen Zhang, Fangpei Li, Wenbo Peng, Quanzhe Zhu, Yongning He

**Affiliations:** 1 School of Microelectronics, Xi’an Jiaotong University, Xi’an 710049, China; 2 The Key Lab of Micro-Nano Electronics and System Integration of Xi’an City, Xi’an 710049, China; 3 Shaanxi Advanced Semiconductor Technology Center Co., Ltd., Xi’an 710077, China

**Keywords:** CuO/ZnO heterojunction, CuO thin film, photodetector, pyro-phototronic effect

## Abstract

The combination of ZnO with narrow bandgap materials such as CuO is now a common method to synthesize high-performance optoelectronic devices. This study focuses on optimizing the performance of p-CuO/n-ZnO heterojunction pyroelectric photodetectors, fabricated through magnetron sputtering, by leveraging the pyro-phototronic effect. The devices’ photoresponse to UV (365 nm) and visible (405 nm) lasers is thoroughly examined. The results show that when the device performance is regulated by adjusting the three parameters—sputtering power, sputtering time, and sputtering oxygen–argon ratio—the optimal sputtering parameters should be as follows: sputtering power of 120 W, sputtering time of 15 min, and sputtering oxygen–argon ratio of 1:3. With the optimal sputtering parameters, the maximum responsivity of the pyroelectric effect and the traditional photovoltaic effect $R_{\mathrm{pyro}+\mathrm{photo}}$ of the detector is 4.7 times that under the basic parameters, and the maximum responsivity of the traditional photovoltaic effect $R_{\mathrm{photo}}$ is also 5.9 times that under the basic parameters. This study not only showcases the extensive potential of the pyro-phototronic effect in enhancing heterojunction photodetectors for high-performance photodetection but also provides some ideas for fabricating high-performance photodetectors.

## 1. Introduction

Metal oxide semiconductor photodetectors have garnered significant attention in recent decades [1,2,3,4]. Among the various types of metal oxide heterojunction photodetectors, CuO/ZnO heterojunction photodetectors, which are wide-bandgap semiconductor-based photodetectors, have been extensively studied due to their excellent photocatalytic properties [5,6,7] and ultraviolet (UV) detection performance [8,9,10]. Current research endeavors focus on improving the performance of these photodetectors [11], including employing photochemical methods to enhance UV detection capabilities [12,13,14], coupling piezo-phototronic effects [15,16,17], and utilizing hydrothermal methods to grow ZnO-branched nanowires on CuO nanowires [18]. The pyroelectric effect is an inherent property of ZnO crystals due to their asymmetric crystal structure. When ZnO nanomaterials are exposed to periodic laser irradiation or undergo significant temperature changes, pyroelectric polarization charges are generated at both ends of the ZnO nanomaterials. The generated pyroelectric polarization charges play a key role in regulating the generation, transportation, recombination, and separation of photogenerated carriers at the heterojunction interface, leading to a phenomenon referred to as the pyro-phototronic effect. Reports indicate that the pyro-phototronic effect in ZnO nanomaterials can be harnessed to modulate the photoresponse current and photodetectivity in optoelectronic devices, including semiconductor heterojunctions [19]. The photothermal conversion in ZnO plays a significant role in the pyroelectric effect during light exposure. Consequently, this pyroelectric effect is leveraged to develop ZnO photodetectors, effectively combining both the pyroelectric and photoexcitation effects [20]. Researchers have utilized various p-type materials in combination with ZnO [21,22,23], designed different core–shell device geometries and sizes [24,25,26], and engineered the surfaces/interfaces of heterojunction devices [27] to achieve pyroelectric photodetection. In addition, the application of anisotropic chemical etching to modify the surface morphology of p-type materials, in conjunction with the pyro-phototropic effect, has previously been investigated by our group as a method to enhance the performance of pyramid-structured ZnO heterojunction photodetectors, with a focus on the influences of surface morphology [28].

Although ZnO-based photodetectors primarily respond to UV light due to their wide bandgap [29,30,31,32], it is also possible to realize broadband photodetectors by utilizing ZnO nanostructures through doping or compositing with other materials [33,34,35]. ZnO functions as an n-type semiconductor in heterojunctions, thus establishing PN junctions with various p-type materials, including Si, GaN, and CuO, which results in a dramatic improvement in the performance of photovoltaic-type photodetectors [36]. Among these p-type materials, CuO is a representative narrow-bandgap metal oxide that shares similarities with ZnO in terms of ionic radius, owns a low heterogeneous crystalline lattice mismatch, and allows for ease of fabrication of high-quality devices. CuO is classified as a direct bandgap semiconductor, possessing a narrow forbidden bandwidth of 1.72 eV, and demonstrates a broad spectrum of absorption characteristics [37]. Recent advancements in research on p-CuO/n-ZnO heterojunctions have progressed significantly and rapidly. In 2020, single CuO-ZnO core–shell radial heterojunction nanowire arrays were successfully fabricated. The preparation of these arrays commenced with the synthesis of CuO nanowire arrays, which served as p-type cores. These were produced through thermal oxidation in an air environment. Following this, ZnO thin films were deposited as n-type shells on the CuO nanowire surfaces via radio-frequency magnetron sputtering [38]. In 2023, the ZnO/CuO nanocomposite was successfully synthesized using a straightforward co-precipitation method, and the band gap energy and transmittance were found to be increased compared with the pure material, as shown by XPS analysis, which demonstrated its promising application in optoelectronics [39].

In p-CuO/n-ZnO heterojunction pyroelectric photodetectors, altering the parameters of CuO modifies the film characteristics, which in turn influences the overall carrier transport process of the device and ultimately affects its performance. However, there is limited research on the effectiveness of magnetron sputtering in regulating the performance of p-CuO/n-ZnO heterojunction photodetectors. While ZnO exhibits the pyroelectric effect due to its asymmetric structure, it is crucial to consider the role of CuO in p-CuO/n-ZnO heterojunction pyroelectric photodetectors. The pyroelectric potential induced by light can modulate the internal electric field at the device’s contact interface, thereby influencing the carrier transport. The separation and transport of these carriers lead to the pyroelectric current. When the surface temperature of the device changes, the pyroelectric effect of ZnO causes charge accumulation at the heterojunction. Since CuO influences the electric field distribution at the heterojunction interface and hence the effects of pyroelectric polarization charges generated inside ZnO, thereby affecting both the intensity and response speed of the pyroelectric effect. Furthermore, charge transfer occurs between CuO (as a p-type material) and ZnO [39], which in turn indirectly regulates the pyroelectric signal response of ZnO.

In this study, a p-CuO/n-ZnO heterojunction pyroelectric photodetector was fabricated using magnetron sputtering. The process involved the sequential deposition of CuO, ZnO, and AZO onto an ITO glass substrate. The optical response of the device to 365 nm and 405 nm lasers in the ultraviolet and visible wavelength ranges was systematically investigated by varying light source power levels. Incorporating the pyro-phototronic effect significantly enhanced the photocurrent and responsivity by more than an order of magnitude. To further optimize device performance, three key sputtering parameters of CuO—sputtering power, sputtering time, and sputtering oxygen–argon ratio—were selected for investigation. The optimal values for these parameters were identified, and their effects on the device’s photoresponse and characteristics were analyzed. The role of each parameter in enhancing device performance was further discussed using a control–variable method.

## 2. Experiment Section

### 2.1. Device Fabrication Process

Using magnetron sputtering, we fabricated CuO/ZnO heterojunction pyroelectric photodetectors on ITO (Indium Tin Oxide) glass substrates. First, the ITO conductive glass (square resistance < 10 ohm/sq, transmittance ≥ 83%) was selected as the substrate and subjected to ultrasonic cleaning with acetone, anhydrous ethanol, and deionized water for 5 min each. Subsequently, a portion of the substrate area was covered with adhesive tape to function as the bottom electrode of the detector. CuO films with varying morphologies were then deposited onto the ITO glass substrate via RF magnetron sputtering (GJP-450) at room temperature. Next, ZnO targets were employed to deposit a series of ZnO films using magnetron sputtering (GJP-450) with a sputtering time of 10 min, a sputtering power of 120 W, and an oxygen–argon ratio of 1:2, followed by the deposition of AZO at room temperature (for 20 min). AZO was utilized as the detector’s top electrode. The test wires were then connected on both the AZO film as the top electrode contact and the ITO as the bottom electrode contact using silver paste. The effective area of the device is 5 mm × 10 mm.

### 2.2. Characterization of Materials and Measurements of Optical and Electrical Properties

The electrical signals from the devices were carefully measured and documented using a dual-channel precision source/measurement unit (B2902A, Keysight, Santa Rosa, CA, USA). The microstructures of the p-type CuO, n-type ZnO, and AZO in the photodetectors were analyzed using scanning electron microscopy (SEM) (GeminiSEM 500, ZEISS, Oberkochen, Germany). Optical input was provided by a handheld UV analyzer (WFH-204B, Shanghai Xu Chang, Shanghai, China) at a wavelength of 365 nm, as well as by a laser operating at a wavelength of 405 nm (M-16A405-100-GLX, MTO-laser, Guangzhou, China). For the 365 nm wavelength, the optical power density was regulated by adjusting the relative position of the light source to the device. In contrast, for the 405 nm wavelength, optical power density was controlled via a continuously variable filter (OMMB-NDFC50, Zolix, Xi’an, China).

## 3. Result and Discussion

### 3.1. Basic Parameter-Based Device Performance

**Figure 1:**
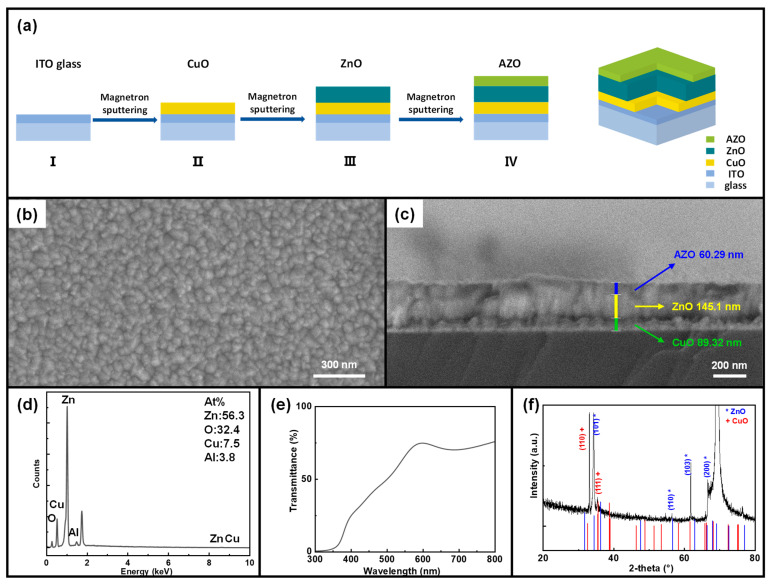
(**a**) Schematic representation of the device fabrication process. (**b**) Top-view scanning electron microscopy (SEM) image of the device, highlighting essential parameters. (**c**) Side-view scanning electron microscopy (SEM) image of the device, illustrating key parameters of each layer. (**d**) EDS spectrum of p-CuO/n-ZnO heterojunction pyro-phototronic photodetector with basic parameters. (**e**) Transmission spectrum of p-CuO/n-ZnO heterojunction with basic parameters. (**f**) XRD pattern of p-CuO/n-ZnO heterojunction with basic parameters.

The fabrication process of a CuO/ZnO heterojunction pyroelectric photodetector on an ITO glass substrate is illustrated in Figure 1a. Figure 1b,c present the top and side views of the CuO devices, respectively. The SEM images indicate that the crystal particles on the surface of the films prepared at room temperature are exceptionally small, with grain sizes in the nanometer scale. The demarcation lines between the p-CuO, n-ZnO, and AZO layers are distinct. The thicknesses measured are approximately 60 nm for the AZO film, 145 nm for the ZnO film, and 89 nm for the bottom CuO thin film. Additionally, AFM test images in Supplementary Material Figure S20 present the surface heights and contact potentials of CuO, CuO-ZnO, and CuO-ZnO-AZO. It can be observed that the surface topography of the fabricated films is smooth, indicating improved surface growth. Figure 1d presents the EDS spectral analysis of the device under basic parameters, which indicates that ZnO has the highest proportion, followed by CuO, and then AZO. This result is consistent with the layer thickness measurements obtained in Figure 1c, further validating the accuracy and reliability of the data. The transmission spectrum of the whole device is shown in Figure 1e, showing that the whole device exhibits high transmittance, ranging from approximately 40% to 70% across wavelengths spanning from about 450 nm to 800 nm. This indicates that the whole device has a low absorption in this wavelength range. The transmission spectrum of CuO/ZnO heterojunction produced by magnetron sputtering is consistent with the experimental findings [8,40,41]. Finally, Figure 1f presents the XRD images of the whole device, with the major orientations corresponding to the XRD peaks of ZnO and CuO as clearly labeled. These results indicate that the whole device exhibits distinctive peaks in the lattice plane families of (101) corresponding to the ZnO and (110) corresponding to the CuO. The deposition was conducted with a sputtering power of 100 W, a duration of 30 min, and an oxygen–argon ratio of 1:3 (JCPDS No. 05-0661, JCPDS No. 36-1451).

**Figure 2:**
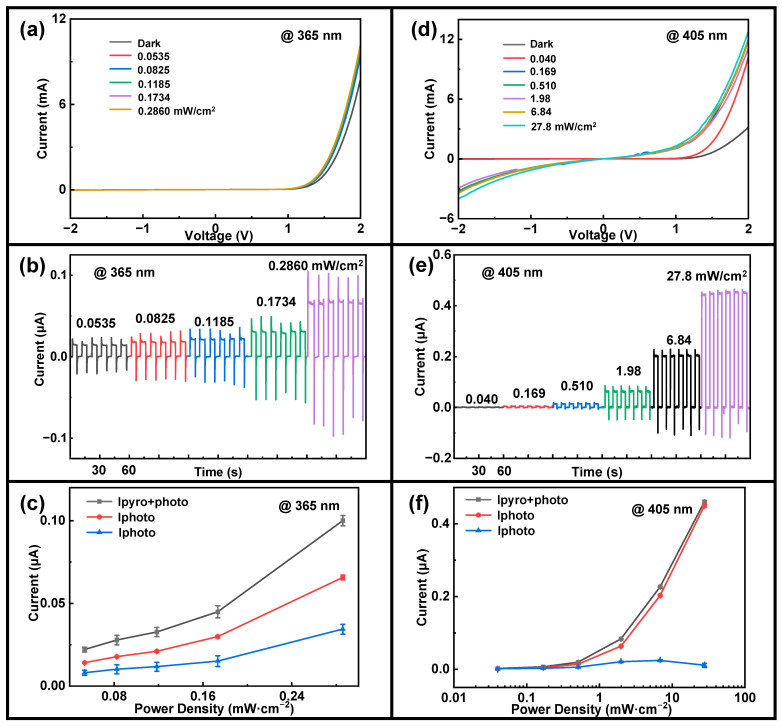
Baseline parameters of the device under illumination with a 365 nm laser at varying optical power densities: (**a**) I-V characteristics, (**b**) I-t transient response, and (**c**) current components ($I_{\mathrm{pyro}+\mathrm{photo}}$, $I_{\mathrm{photo}}$, and $I_{\mathrm{pyro}}$) at each optical power density. Baseline parameters of the device under illumination with a 405 nm laser at varying optical power densities: (**d**) I-V characteristics, (**e**) I-t transient response, and (**f**) current components ($I_{\mathrm{pyro}+\mathrm{photo}}$, $I_{\mathrm{photo}}$, and $I_{\mathrm{pyro}}$) at each optical power density.

Figure 2a,d depict the current–voltage (I-V) characteristics of the p-CuO/n-ZnO heterojunction pyroelectric photodetector under 365 nm and 405 nm illumination. The device showed a strong photoresponse under forward bias, with the forward saturation current increasing consistently with light power density. The current at +2V bias increases dramatically from ~7.7 mA under dark conditions to ~10.2 mA under 0.286 mW/cm^2^ 365 nm UV illumination and from ~3.1 mA under dark conditions to ~12.7 mA under 27.8 mW/cm^2^ 405 nm illumination, respectively. These results demonstrate that the p-CuO/n-ZnO heterojunction exhibits good rectification behavior, suggesting effective electrical contact between CuO and ZnO.

Additionally, for a detailed transient characteristic analysis, the device was periodically illuminated by 365 nm and 405 nm lasers under zero bias, as shown in Figure 2b,e. When a regularly switched laser illuminates the p-CuO/n-ZnO heterojunction pyroelectric photodetector, the device’s response demonstrates a classic four-step pyroelectric response [42]. Initially, the photodetector resides in a dark state, characterized by a minimal dark current $I_{\mathrm{dark}}$ within the device. Upon irradiation with laser light, a photovoltaic-induced current $I_{\mathrm{photo}}$ is generated within the circuit. Additionally, the temperature of ZnO experiences an instantaneous increase, resulting in a positive rate of temperature change (dT/dt>0). The temperature variation generates a negative pyroelectric polarization potential at the p-CuO/n-ZnO heterojunction interface. This pyroelectric voltage $V_{\mathrm{pyro}}$ across the ZnO aligns with $I_{\mathrm{photo}}$, enabling the current to rapidly peak from the initial dark current, which includes both steady-state and pyroelectric photocurrents. Consequently, $I_{\mathrm{photo}}$ continues to increase until it reaches the peak value $I_{\mathrm{pyro}+\mathrm{photo}}$. Under continuous illumination, the temperature of ZnO gradually rises until it reaches saturation, at which point dT/dt=0. Then, the variations in temperature and the pyroelectric effect in ZnO progressively diminish, ultimately leaving only the steady-state photocurrent generated by photoexcitation. Consequently, the current decreases from its peak value $I_{\mathrm{pyro}+\mathrm{photo}}$ to $I_{\mathrm{photo}}$. When the illumination is abruptly turned off, the temperature of ZnO experiences an instantaneous decline, leading to a condition where dT/dt<0. This phenomenon induces a pyroelectric polarization potential that is contrary to the one generated during illumination, thereby limiting carrier transport within the heterojunction. As a result, the photocurrent experiences an immediate drop, leading to a reverse spike in $I_{\mathrm{pyro}}$ due to the opposing pyroelectric potential. Figure S23a,b show the schematic energy band diagram of the device when light is turned on and off, clearly demonstrating the effects of the pyroelectric effect on the energy band diagram.

When the light source is turned off and the device reverts to its dark state, the temperature of ZnO returns to room temperature and stabilizes, at which point dT/dt=0. Consequently, the pyroelectric effect in ZnO diminishes once again, leading to a return of the current to its initial steady-state dark current $I_{\mathrm{dark}}$ [43]. The dark current level is on the order of 10^−9^ A. In contrast to similar devices featuring an ITO-CuO-ZnO-ITO structure with a CuO sputtering power of 50 W, a sputtering time of 30 min, and an oxygen–argon ratio of 1:3, which typically display dark current levels on the order of 10^−3^ A, this constitutes a significant enhancement in performance [28]. The irradiation of the device was controlled by moving a black opaque cardboard, combined with a stopwatch to change the light on/off state every 5 s, achieving periodic irradiation of the device with a whole cycle of 60 s for each light power density. The power range of the 365 nm laser was measured to be 5.35 × 10^−2^–2.86 × 10^−1^ mW/cm^2^, while the power range of the 405 nm laser was recorded as 4.0 × 10^−2^–27.8 mW/cm^2^. According to Figure 2b,e, it is evident that at all light power densities, each light pulse exhibits a four-stage current response induced by the pyroelectric effect. The negative spike observed in the image, which represents the pyroelectric current, can be expressed as $I_{\mathrm{pyro}}=\eta P_{c}A(\frac{\mathrm{dT}}{\mathrm{dt}})$, where η denotes the light absorption coefficient, A represents the electrode area of the device, $P_{c}$ signifies the pyroelectric coefficient, and $\frac{\mathrm{dT}}{\mathrm{dt}}$ is the rate at which the temperature of the device changes. It can be seen that I_pyro_ is proportional to $\frac{\mathrm{dT}}{\mathrm{dt}}$. In Figure 2b,e, both the positive current spike I_pyro+photo_ and the negative current spike I_pyro_ exhibit an increase with rising light power density. This phenomenon occurs because, as illumination intensifies, the light power density escalates, thereby enhancing the laser’s heating capability on the device, which in turn increases $\frac{\mathrm{dT}}{\mathrm{dt}}$, leading to an increase in pyroelectric current intensity. For this detector under zero bias, under 365 nm wavelength laser irradiation, I_pyro+photo_ increases from 0.02199 μA (0.0536 mW/cm^2^) to 0.10009 μA (0.286 mW/cm^2^) with increasing laser power density, and $I_{\mathrm{pyro}}$ increases from 0.00797 μA (0.0536 mW/cm^2^) to 0.03439 μA (0.286 mW/cm^2^). Under 405 nm wavelength laser irradiation, I_pyro+photo_ also increases rapidly with increasing laser power density, from 0.00165 μA (0.04 mW/cm^2^) to 0.46011 μA (27.8 mW/cm^2^), and I_pyro_ increases from 8.04 × 10^−4^ μA (0.04 mW/cm^2^) to 0.03439 μA (27.8 mW/cm^2^). Additionally, as demonstrated in Figure S1a,b, the ratio of I_pyro_/I_photo_ decreases with increasing light power density for a fixed laser wavelength. This decrease is more pronounced at 405 nm compared to 365 nm. As a consequence, the total current I_pyro+photo_ is mainly contributed by the photovoltaic effect.

**Figure 3:**
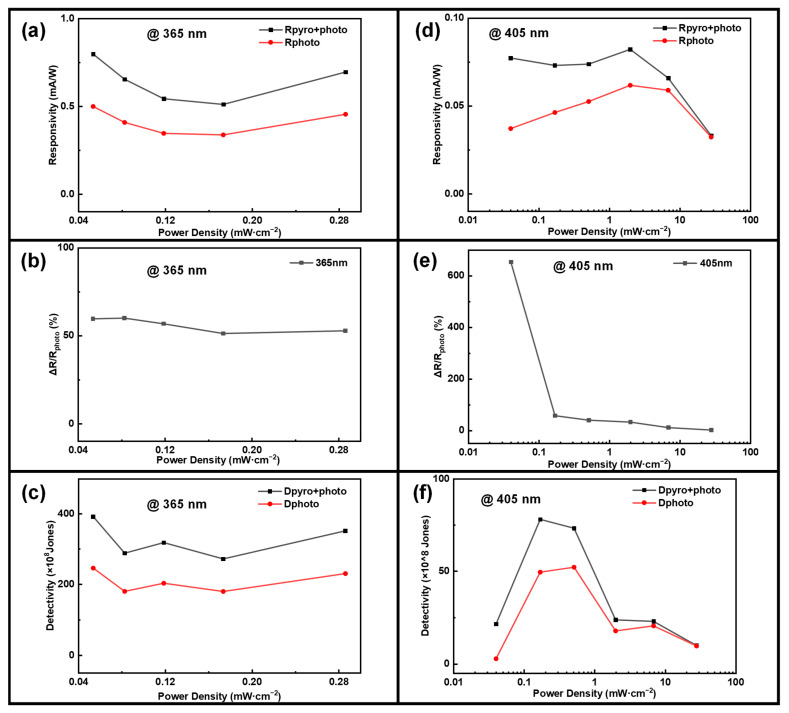
(**a**) The responsivity as a function of power density. (**b**) The maximum gain of responsivity $\Delta R/R_{\mathrm{photo}}$ as a function of power density. (**c**) The detectivity of the basic parameter device under 365 nm laser irradiation as a function of power density. (**d**) Responsivity plotted against power density. (**e**) The maximum gain in responsivity $\Delta R/R_{\mathrm{photo}}$ versus power density. (**f**) The detectivity of the basic parameter device under 405 nm laser irradiation as a function of power density.

To demonstrate the improvement in photodetection performance facilitated by the pyro-phototronic effect, Figure 2c,f present the calculated and plotted variations of three distinct currents in relation to optical power density. The total photocurrent generated by the pyro-phototronic effect is denoted as I_pyro+photo_, while I_photo_ represents the photocurrent produced via the traditional photovoltaic effect, and I_pyro_ corresponds to the photocurrent arising from the pyroelectric effect. As illustrated in Figure 2c,f, under illumination from laser light sources of varying wavelengths and power densities, the pyro-phototronic effect enhances the photodetector’s current from I_photo_ to I_pyro+photo_. As illumination intensity increases, I_pyro_ improves only slightly, while I_pyro+photo_ and I_photo_ improve significantly. Meanwhile, according to the formula for responsivity [44]. $R=\frac{I_{\mathrm{photo}}-I_{\mathrm{dark}}}{\mathrm{pS}}$, where p is the optical power density and S represents the effective area of the light spot, the unit of responsivity is mA/W. Figure 3a,b illustrate the variations in responsivity, $R_{\mathrm{pyro}+\mathrm{photo}}$ and $R_{\mathrm{photo}}$, as a function of increasing optical power density. The results indicate that under irradiation with lasers at 365 nm and 405 nm, the responsivity diminishes as the optical power density increases. It is noteworthy that at each level of optical power density, the responsivity $R_{\mathrm{pyro}+\mathrm{photo}}$ is significantly higher than $R_{\mathrm{photo}}$ due to the contribution of the pyroelectric effect.

In Figure 3a, the responsivity at 365 nm exhibits a non-monotonic trend, initially decreasing and then increasing as the power density rises. This behavior results from the interplay between two competing mechanisms. At lower power densities, the responsivity decreases due to the saturation of photogenerated carriers, as increased light intensity causes a saturation effect that limits the photoresponse. However, as the power density further increases, the temperature gradient induced by higher light intensity enhances the pyroelectric effect, contributing additional photocurrent. This enhanced pyroelectric contribution leads to a subsequent increase in responsivity at higher power densities. In contrast, Figure 3d shows a different trend for responsivity at 405 nm due to the less pronounced pyroelectric effect at this longer wavelength compared to 365 nm. The responsivity at 405 nm initially increases with power density, primarily due to higher photogeneration efficiency. At low power densities, increased light absorption generates a larger number of carriers, enhancing the responsivity. However, at higher power densities, carrier recombination effects become significant, reducing the number of free carriers available for conduction and leading to a decrease in responsivity.

Additionally, according to the formula for detectivity $D^{*}$ of the photodetector [43], under 365 nm and 405 nm lasers, calculated as $D^{*}=\frac{R}{\sqrt{2qJ_{d}}}$, where $J_{d}$ is the dark current density $J_{d}=\frac{I_{\mathrm{dark}}}{S}$, and q is the charge quantity, the unit of detectivity is Jones. The relevant plots are presented in Figure 3c,f. Detectivity is a crucial parameter for assessing the capability of a photodetector to identify weak light signals. It defines the minimum detectable signal by taking into account the dark current as the primary source of noise. From Figure 3c,f, it is evident that detectivity exhibits a similar trend to responsivity. At each optical power density, the pyro-phototronic effect has brought dramatic improvements compared to the photovoltaic effect-induced R_photo_ and D*_photo_. Under 365 nm laser, R has been improved from ~0.45 mA/W to ~0.64 mA/W (by averaging over all the power densities) and D from ~208 × 10^8^ Jones to ~324 × 10^8^ Jones (by averaging over all the power densities). For the 405 nm laser, R has been improved from ~0.063 mA/W to ~0.074 mA/W and D from ~27 × 10^8^ Jones to ~33 × 10^8^ Jones. $D_{\mathrm{pyro}+\mathrm{photo}}^{*}$ significantly exceeds $D_{\mathrm{photo}}^{*}$ due to the pyro-phototronic effect. The calculation of the maximum gain in photodetector responsivity relative to light power density is presented herein, $\frac{\Delta R}{R_{\mathrm{photo}}}=\frac{R_{\mathrm{pyro}}}{R_{\mathrm{photo}}}$, as depicted in Figure 3b,e. It is evident that under exposure to a 365 nm laser at 8.25 × 10^−1^ mW/cm^2^, $R_{\mathrm{pyro}}$ exceeds $R_{\mathrm{photo}}$ by a factor of 60, while exposure to a 405 nm laser at 4.0 × 10^−2^ mW/cm^2^ results in $R_{\mathrm{pyro}}$ exceeding $R_{\mathrm{photo}}$ by a factor of 108.

In Figure 3b, the responsivity gain at 365 nm follows a distinct pattern: it initially increases and then decreases with increasing power density. At lower power densities, the pyroelectric contribution to the total responsivity is relatively large compared to the baseline photoresponsivity, resulting in a higher gain. As the power density increases, the dominant contribution shifts from the pyroelectric effect to photogenerated carriers. Consequently, the relative enhancement provided by the pyroelectric effect diminishes, causing the overall gain to decrease. Under illumination at 405 nm, both ZnO and CuO are capable of generating electron–hole pairs that contribute to the photocurrent, resulting in a high maximum gain. Due to the significant absorption coefficient of ZnO in the ultraviolet region, whereas CuO exhibits minimal absorption of ultraviolet light for electron–hole pair generation, the maximum gain under 365 nm illumination is relatively low. The maximum gain in detectivity, $\frac{\Delta D}{D_{\mathrm{photo}}}=\frac{D_{\mathrm{pyro}}}{D_{\mathrm{photo}}}$, calculated with varying light power density, is shown in Figure S1b,d. It is evident that under both 365 nm and 405 nm laser illuminations, the trend aligns consistently with the maximum gain in responsivity, indicating that the pyroelectric effect holds significant potential for enhancing photodetector performance. Response time is also a crucial parameter of photodetectors. The response time is categorized into rise time and fall time. For the devices fabricated by us, the rise time refers to the period during which $I_{\mathrm{pyro}+\mathrm{photo}}$ of the device ascends from 10% to 90% when the external light is turned on. Fall time is the time when the device drops from 90% of the difference between $I_{\mathrm{photo}}$ and reverse $I_{\mathrm{photo}}$ to 10% when the external light is turned off.

### 3.2. Sputtering Power

To investigate the influence of the CuO layer in p-CuO/n-ZnO heterojunction pyroelectric photodetectors, different p-CuO thin films were deposited under various sputtering powers while keeping other conditions constant. The same transient response test was then conducted on the resulting photodetectors. Experiments were conducted using three sputtering powers (100 W, 120 W, and 140 W) for CuO deposition, maintaining consistent sputtering conditions otherwise.

**Figure 4:**
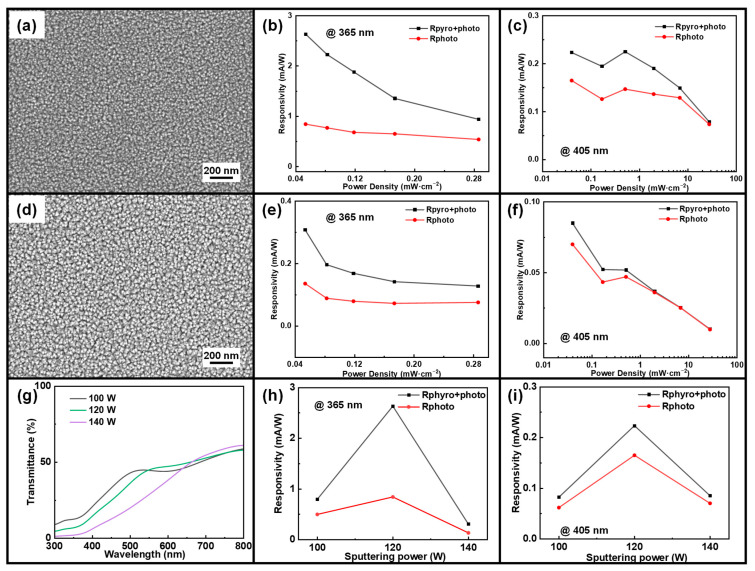
(**a**) SEM image of CuO at a sputtering power of 120 W. Responsivity of the device at 120 W sputtering power as a function of light intensity at (**b**) 365 nm and (**c**) 405 nm. (**d**) SEM image of CuO at a sputtering power of 140 W. Responsivity of the device at 140 W sputtering power as a function of light intensity at (**e**) 365 nm and (**f**) 405 nm. (**g**) Transmission spectra of the devices at three different sputtering powers. Responsivity of the devices at three different sputtering powers as a function of light intensity at (**h**) 365 nm and (**i**) 405 nm.

The top SEM images in Figure 4a,d depict CuO thin films on silicon wafers sputtered at 120 W and 140 W for 30 min, with an oxygen–argon ratio of 1:3. The CuO film at 120 W sputtering power is denser than the film at 140 W sputtering power. As the sputtering power is increased, distinct variations in the surface morphology of the deposited films are observed. Figure S6a,d show the cross-sectional SEM images of CuO films under 120 W and 140 W sputtering power. All cross-sectional SEM images show the well-sputtered CuO films, but the thickness of the films increases with increasing sputtering power. Figure S6b,e show the EDS mappings of CuO films under 120 W and 140 W sputtering power. Combining the two XRD images of Figure S6c,f, we can see that the positions of the main peaks of CuO are similar under different sputtering powers, indicating that the crystalline phases of the films have not changed significantly and the characteristic crystalline phases of CuO are still preserved. The main diffraction peaks of the 120 W sample are relatively sharper, and the peak widths are narrower, indicating that the films are more crystallized under 120 W sputtering power. Figure 4g presents the transmission spectra of CuO films at various sputtering powers. The data indicate a notable decrease in transmittance within the shorter wavelength range (300–500 nm) as the sputtering power increases. Furthermore, higher sputtering powers result in improved transmittance at higher wavelengths and reduced transmittance at lower wavelengths. Furthermore, Figure 4g shows that the absorption edge of the CuO film sputtered at 140 W exhibits the slowest response, indicating the poorest crystallinity. Combining this with the SEM images, it can be concluded that higher sputtering power increases the thickness, thereby affecting the light transmission properties in different wavelength ranges.

For the p-CuO/n-ZnO heterojunction pyroelectric photodetectors under sputtering powers of 120 W and 140 W, the I-V characteristics of the detector under 120 W sputtering power under 365 nm and 405 nm illumination were first tested and plotted in Figure S2a,d. The device demonstrates favorable forward bias photoresponse characteristics, with the forward saturation current progressively increasing with rising light power density at constant forward bias voltage. The I-t characteristics under both illuminations are presented in Figure S2b,e. These measurements were conducted under the same testing conditions as those applied to the device at 100 W sputtering power. The device demonstrates the four-stage current response due to the pyroelectric photoelectric coupling effect in each cycle, indicating robust I-t characteristics. Furthermore, the photocurrent response gradually increases with the increase of light intensity. The I-V characteristics under 365 nm and 405 nm laser illumination at 120 W sputtering power are also measured and plotted in Figure S2a,d, showing that the device still has good forward saturation photocurrent at this sputtering power, while the reverse photocurrent is close to 0. Figure S2c,f shows the variation of current with light power density under the two types of laser illumination. Figure S3 shows the maximum responsivity gain, detectivity, and maximum detectivity gain of the device at 120 W sputtering power under periodic irradiation of the two types of lasers as a function of light power density. The current–voltage, current–time, and current–light power density plots of the detector under 365 nm and 405 nm laser illumination at 140 W sputtering power are shown in Figure S4. The maximum responsivity gain, detectivity, and maximum detectivity gain under the two types of illumination are shown in Figure S5. Through comprehensive comparison, we found that whether the device is fabricated under 120 W or 140 W sputtering power, it can be seen from Figure S2a,d that it has good rectification behavior, and it can be seen that the pyro-phototronic effect has a significant improvement on the devices’ performances.

Figure 4b,c show the responsivity of the photodetector under 365 nm and 405 nm lasers at sputtering powers of 120 W and 140 W, respectively. Under both laser wavelengths, it can be observed that $R_{\mathrm{pyro}+\mathrm{photo}}$ and $R_{\mathrm{photo}}$ decrease with increasing light intensity. However, at each light power density, the responsivity $R_{\mathrm{pyro}+\mathrm{photo}}$ is higher than $R_{\mathrm{photo}}$ due to the pyroelectric effect. For the sputtering power of 120 W, R has been improved from ~0.7 mA/W to ~1.8 mA/W under 365 nm laser and from ~0.13 mA/W to ~0.18 mA/W under 405 nm laser. For the sputtering power of 140 W, R has been improved from ~0.09 mA/W to ~0.19 mA/W under a 365 nm laser and from ~0.039 mA/W to ~0.044 mA/W under a 405 nm laser. Hence, we can see that at 120 W sputtering power, the pyroelectric current has the largest overall current enhancement effect on the device, and the pyro-phototronic effect is the most significant. Figure 4h,i illustrate the variation in responsivity $R_{\mathrm{pyro}+\mathrm{photo}}$ and $R_{\mathrm{photo}}$ as a function of sputtering power under 365 nm and 405 nm lasers, respectively. The photodetector exhibits the highest responsivity at a sputtering power of 120 W for both laser wavelengths, indicating optimal performance at this power level.

The comparison of response times under different sputtering powers (100 W, 120 W, and 140 W) at two illumination wavelengths is presented in Figures S21a,d and S22a,d. At the lower sputtering power of 100 W, the side-view SEM image in Figure 1c reveals insufficient growth of the CuO thin film, resulting in a thin, non-uniform layer with poor surface coverage and inadequate thickness. This observation is further supported by the AFM images in Figure S20, which compare the bottom CuO thin films with CuO/ZnO and CuO-ZnO-AZO heterojunction devices. The CuO film exhibits a surface roughness of approximately 10 nm, characterized by uneven surfaces and distinct, isolated grain structures. In contrast, increasing the sputtering power to 140 W significantly enhances the CuO film’s thickness, as shown in Figure S6. However, higher sputtering power introduces excessive defects and internal stress [45,46,47,48]. These effects are evident in the device’s performance, particularly in the prolonged response times observed at higher sputtering power, as shown in Figures S21 and S22. The side-view SEM image in Figure S6 indicates that the CuO film thickness increases by approximately 20 nm compared to that at 120 W. This increased thickness hinders the transport and separation of photogenerated carriers, thereby reducing the device’s responsivity to some degree.

Combining the obtained characterization results and the test analysis of the device under two lasers, the sputtering power of 120 W was identified as optimal for the p-CuO device. Subsequent experiments were conducted using this optimized sputtering power to ensure enhanced device performance.

### 3.3. Sputtering Time

**Figure 5:**
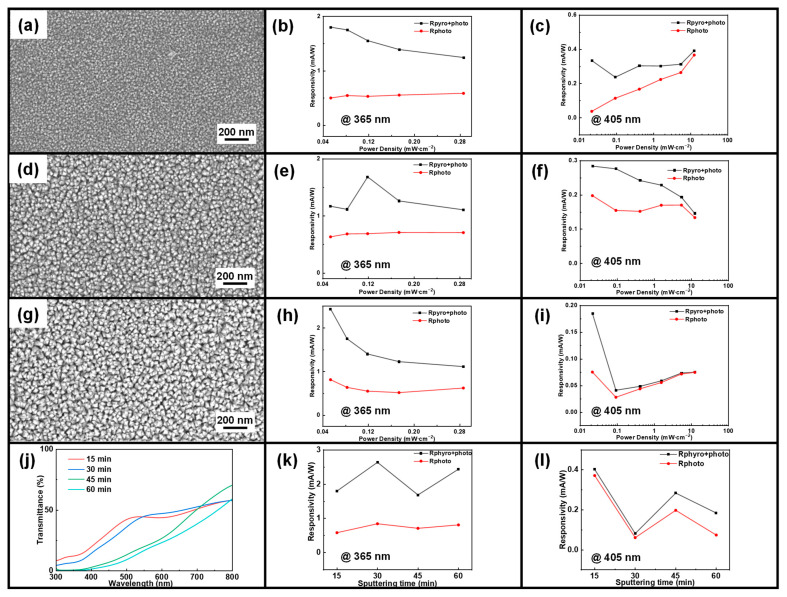
(**a**) SEM image of CuO after 15 min of sputtering; device responsivity under (**b**) 365 nm and (**c**) 405 nm illumination as a function of light intensity after 15 min of sputtering. (**d**) SEM image of CuO after 45 min of sputtering; device responsivity under (**e**) 365 nm and (**f**) 405 nm illumination as a function of light intensity after 45 min of sputtering. (**g**) SEM image of CuO after 60 min of sputtering; device responsivity under (**h**) 365 nm and (**i**) 405 nm illumination as a function of light intensity after 60 min of sputtering; and (**j**) transmission spectra of devices with four different sputtering durations; device responsivity under (**k**) 365 nm and (**l**) 405 nm illumination as a function of light intensity for four different sputtering durations.

The sputtering power was fixed at 120 W. Experiments were then performed for four different sputtering times (15 min, 30 min, 45 min, and 60 min) with all other conditions unchanged. Figure 5a,d,g, respectively, show the top SEM images of CuO films deposited on silicon wafers with sputtering times of 15 min, 45 min, and 60 min. It can be observed that as the sputtering time increases, the surface of the obtained films becomes rougher. The CuO film sputtered for 15 min is denser compared to the films obtained with other sputtering times. Figure S13 shows the cross-sectional SEM images, EDS mappings, and XRD images of CuO films sputtered on silicon wafers for 15 min, 45 min, and 60 min. The cross-sectional SEM images reveal that while the CuO film’s thickness increases with sputtering time, the growth becomes increasingly disordered with extended deposition duration. Thinner films demonstrate greater uniformity and fewer structural defects. In contrast, extended sputtering times facilitate the formation of columnar structures or larger grains [48], which could adversely impact carrier transport and lower device responsivity. The EDS mappings further support these observations, indicating that the sample deposited for 15 min exhibits the most uniform distribution of Cu and O, suggesting better stoichiometric control and consistent film coverage. In contrast, longer sputtering times lead to material inconsistencies and aggregation, with evidence of cluster formation in the EDS images. These findings are further supported by the XRD patterns. The 15-min sample displays sharp, well-defined peaks, reflecting superior crystallinity. In comparison, the 45-min and 60-min samples show broader peaks and increased background noise, indicating diminished crystallinity and the presence of amorphous or polycrystalline phases caused by excessive deposition. Figure 5j shows the transmission spectra of CuO films for different sputtering times. For shorter sputtering times (15 min and 30 min), the transmittance increases rapidly. However, for longer sputtering times (45 min and 60 min), the increase in transmittance is more significant. This indicates that longer sputtering times may increase the film density, leading to higher transmittance. It can be seen that at the sputtering time of 15 min, the absorption edge of the CuO film is the sharpest, indicating that the film has the best crystallinity at this sputtering time.

Figures S7, S9, and S11 show the current–voltage (I-V) curves, current–time (I-t) curves, and current–optical power density (I-P) curves of devices at two light wavelengths under sputtering power of 120 W and an oxygen–argon ratio of 1:3, with sputtering times of 15 min, 45 min, and 60 min, respectively. It can be observed that all the devices fabricated at different sputtering times exhibit good rectification characteristics, with current increasing with optical power density under forward bias and approaching zero under reverse bias. At zero bias, all the devices show a four-stage current response under periodic light conditions. Additionally, as optical power density increases, both I_pyro+photo_ and I_photo_ increase significantly, while I_pyro_ remains smaller. This suggests that the photovoltaic current still makes a significant contribution to the total current, which is consistent with our previous findings.

Figure 5b,e,h present the responsivity, $R_{\mathrm{pyro}+\mathrm{photo}}$ and $R_{\mathrm{photo}}$, of photodetectors under 365 nm laser illumination for varying sputtering durations. It is evident that the responsivity generally decreases with increasing light power density, and the pyroelectric effect results in $R_{\mathrm{pyro}+\mathrm{photo}}$ being higher than $R_{\mathrm{photo}}$. For sputtering time of 15 min, R has been improved from ~0.54 mA/W to ~1.55 mA/W. Figure 5c,f,i present the responsivity $R_{\mathrm{pyro}+\mathrm{photo}}$ and $R_{\mathrm{photo}}$ of photodetectors exposed to a 405 nm laser for various sputtering durations. The results are consistent with those obtained under a 365 nm laser, reinforcing the conclusion that the pyroelectric effect enhances device responsivity. For sputtering time of 15 min, R has been improved from ~0.23 mA/W to ~0.38 mA/W. It is very clear that the device response is maximum at 15 min sputtering time. Figure 5k,l illustrate the changes in responsivity $R_{\mathrm{pyro}+\mathrm{photo}}$ and $R_{\mathrm{photo}}$ under 365 nm and 405 nm lasers with varying sputtering times. The data indicate that the photodetector achieves its maximum responsivity under the 405 nm laser at a sputtering time of 15 min. Figures S8, S10, and S12 show the maximum responsivity gain–optical power density curves, detectivity-optical power density curves, and maximum gain detectivity–optical power density curves of devices at two light wavelengths under sputtering power of 120 W and an oxygen–argon ratio of 1:3, with sputtering times of 15 min, 45 min, and 60 min, respectively. It can be seen that the R and D of the device are relatively good, indicating the good electrical characteristics of the device. As the optical power density increases, the Ipyro also gradually increases, and the overall response contribution in the device also gradually increases. The comparison of response times under different sputtering times at two illumination wavelengths is presented in Figures S21b,e, and S22b,e. The EDS mapping of the 15-min sample shows a more uniform Cu and O distribution, indicating better stoichiometric control. For longer sputtering times, the resulting films are thicker, with increased structural irregularities and higher defect density. These issues lead to increased scattering and slower response times, varying sputtering durations.

Therefore, combining the obtained characterization results and the test analysis of the device under two lasers, we conclude that a 15-min sputtering time yields the highest-quality CuO thin film, characterized by minimized structural defects, uniform coverage, and enhanced crystallinity.

### 3.4. Sputtering Oxygen–Argon Ratio

**Figure 6:**
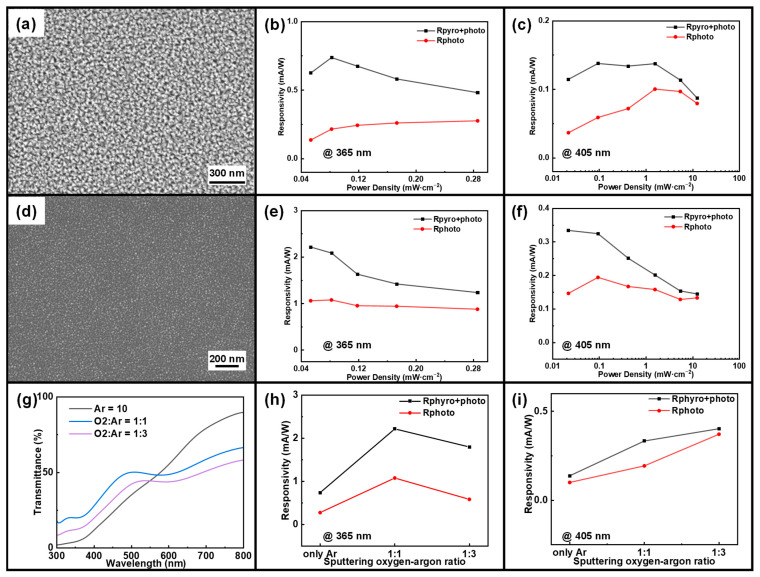
(**a**) SEM image of CuO under pure Ar. Responsivity of the device under pure Ar at (**b**) 365 nm and (**c**) 405 nm as a function of light intensity. (**d**) SEM image of CuO with an O_2_/Ar ratio of 1:1. Responsivity of the device with an O2/Ar ratio of 1:1 at (**e**) 365 nm and (**f**) 405 nm as a function of light intensity. (**g**) Transmission spectra of devices with three different O_2_/Ar sputtering ratios. Responsivity of devices with three different O2/Ar sputtering ratios at (**h**) 365 nm and (**i**) 405 nm as a function of light intensity.

Using a sputtering power of 120 W and a sputtering time of 15 min, experiments were next conducted with three different oxygen–argon ratios: pure Ar, an oxygen–argon ratio of 1:1, and an oxygen–argon ratio of 1:3. Figure 6a,d shows the SEM images of CuO films deposited on silicon wafers at a fixed sputtering power of 120 W and a sputtering time of 15 min, under pure Ar and an oxygen–argon ratio of 1:1, respectively. Figure S18 shows the cross-sectional SEM images, EDS mappings, and XRD images of CuO films sputtered on silicon wafers for three different oxygen–argon ratios. It can be observed that in a pure argon environment, the surface crystal particles are dense and uniformly distributed, with higher roughness. When the oxygen–argon ratio is 1:1, the number of surface crystal particles decreases compared to a pure argon environment, resulting in a sparser distribution and a smoother surface. At an oxygen–argon ratio of 1:3, the number of surface crystal particles decreases further, leading to a more uniform distribution and an even smoother surface. Thus, different gas environments significantly impact the surface morphology of the material. Increasing the oxygen proportion reduces surface particle density and surface roughness. In a pure argon environment, deposited CuO’s surfaces contain dense and rough crystal particles. However, as the oxygen proportion increases, the surface crystal particle density decreases, resulting in a smoother surface. Oxygen effectively inhibits excessive particle growth to a certain extent. The CuO film grown under an oxygen–argon ratio of 1:3 exhibits a moderate and uniform thickness, in contrast to the excessively thick film obtained under pure Ar and the overly thin film grown with an oxygen–argon ratio of 1:1. This moderate thickness ensures optimal film growth and uniformity, while excessively thick films suffer from poor crystallinity and internal stress [47], and overly thin films result in incomplete coverage and reduced quality. EDS mapping results further demonstrate that the oxygen–argon ratio of 1:3 condition yields the most homogeneous distribution of Cu and O, unlike the other conditions, which exhibit either oxygen deficiencies or non-uniform elemental distributions that compromise film integrity. XRD patterns confirm these findings, revealing sharp and well-defined peaks for the oxygen–argon ratio of 1:3, indicative of improved crystallinity. In comparison, films grown under pure Ar exhibit poor crystalline quality or non-stoichiometric phases due to insufficient oxygen supply. Similarly, the oxygen–argon ratio of 1:1 condition leads to suboptimal stoichiometry and crystallinity, attributed to an imbalance in the gas ratio. The response time measurements further confirm that the oxygen–argon ratio of 1:3 condition results in the fastest response, aligning with the superior film quality observed in the SEM and EDS analyses.

Figure 6g illustrates the transmittance spectra of CuO films under varying oxygen–argon sputtering ratios. It is evident that transmittance increases with wavelength across all curves. In the shorter wavelength range (from 300 nm to 500 nm), transmittance remains relatively low, with noticeable differences among different oxygen–argon ratios. Conversely, in the longer wavelength range (from 500 nm to 800 nm), transmittance shows a gradual increase, with values for different oxygen–argon ratios tending to converge. The transmittance is highest in a pure argon environment, indicating that the material’s transmittance performance is best without oxygen. The introduction of oxygen reduces the transmittance of the material, and the transmittance is lowest in the oxygen–argon ratio of 1:3 environment, indicating that a high oxygen ratio has a more significant negative impact on transmittance performance. Regardless of the gas environment, transmittance increases with wavelength, but the introduction of oxygen overall lowers the transmittance.

Figures S14 and S16 present the current–voltage, current–time, and current–light power density graphs of devices sputtered at 120 W for 15 min under pure argon and an oxygen–argon ratio of 1:1, respectively, at two wavelengths. It can be seen that the rectification characteristics of the devices are very different between the pure Ar and the oxygen–argon ratio of 1:1 and not as good as the devices fabricated in the oxygen–argon ratio of 1:3. When periodically illuminated, the four-stage response is still exhibited at zero bias, and it can be seen that the pyroelectric still has an enhancing effect on its light response. Similar to the previous conclusion, as the optical power density on the surface of the device increases, I_pyro+photo_ increases, and I_photo_ occupies the main contribution. Figure 6b,e display the detector responsivity $R_{\mathrm{pyro}+\mathrm{photo}}$ and $R_{\mathrm{photo}}$ under two different oxygen–argon ratios as the light intensity varies with a 365 nm wavelength laser. It is observed that as the power density of the 365 nm laser increases, the responsivity decreases, and the pyroelectric effect becomes notably significant. For different oxygen–argon ratios, R has been improved from ~0.23 mA/W to ~0.62 mA/W (pure Ar) and from ~0.98 mA/W to ~1.72 mA/W (oxygen–argon ratio of 1:1). It can be seen that the pyroelectric effect has a significant enhancement on the overall device responsivity. Figure 6c,f depict the detector’s responsivity versus light intensity under 405 nm laser illumination. R has been improved from ~0.074 mA/W to ~0.12 mA/W (pure Ar) and from ~0.15 mA/W to ~0.24 mA/W (oxygen–argon ratio of 1:1). It is evident that the overall trend mirrors that observed under 365 nm laser illumination, thereby reinforcing our previous conclusions. Figures S15 and S17 show the maximum responsivity gain–light power density, detectivity–light power density, and detectivity maximum gain–light power density graphs of devices sputtered at 120 W for 15 min under pure argon and an oxygen–argon ratio of 1:1, respectively, at two wavelengths. It can be observed that both I_photo_ and I_pyro_ within the device increase as the optical power density rises. However, the maximum gains in responsivity and detectivity decrease with increasing optical power density, suggesting that I_pyro_ increases at a slower rate compared to I_photo_.

Figure 6h,i shows the changes in responsivity $R_{\mathrm{pyro}+\mathrm{photo}}$ and $R_{\mathrm{photo}}$ at 365 nm and 405 nm lasers as the argon–oxygen ratio changes. Firstly, the addition of oxygen significantly enhances the device’s responsivity under both 365 nm and 405 nm lasers, especially when the argon–oxygen ratio is 1:1. Secondly, the responsivity at 365 nm reaches its peak at an oxygen–argon ratio of 1:1, while the responsivity at 405 nm gradually increases with the proportion of oxygen. Furthermore, the sum of the pyroelectric and photoelectric responses ($R_{\mathrm{pyro}+\mathrm{photo}}$) under both laser conditions exceeds the individual photoelectric response ($R_{\mathrm{photo}}$), indicating a significant contribution of the pyroelectric effect to the overall response. The comparison of response times under different sputtering times at two illumination wavelengths is presented in Figures S21c,f and S22c,f. Among the three oxygen–argon ratios tested, the ratio of 1:3 produced the best results. During the sputtering process, oxygen acts as an oxidizing agent. A higher oxygen ratio can result in the formation of a purer CuO film, reducing defects and impurities, which in turn enhances its photoelectric performance. Additionally, a higher oxygen ratio can produce finer and more uniform grains in the film, thereby increasing light absorption and carrier mobility. Furthermore, it can improve the interface quality by reducing interface defects and recombination centers, thereby enhancing carrier separation efficiency.

Therefore, combining the preliminary performance tests and the subsequent characterization results, we can obtain the best performance of the devices fabricated when the sputtered oxygen–argon ratio is 1:3.

### 3.5. Optimized Parameters Device

Combining the previously mentioned studies, we systematically investigated the parameters of magnetron-sputtered CuO thin films. By integrating device performance tests with thin-film characterization results, we concluded that the performance of p-CuO/n-ZnO heterojunction pyroelectric photodetectors can be effectively modulated.

After evaluating the effects of sputtering power, sputtering time, and oxygen–argon ratio on the performance of p-CuO/n-ZnO heterojunction pyro-photovoltaic photodetectors, the optimal parameters for CuO deposition were determined to be a sputtering power of 120 W, a sputtering time of 15 min, and an oxygen–argon ratio of 1:3. Characterization of the optimized detector included SEM top-view and side-view images, EDS mapping, transmittance spectra, and XRD patterns, as illustrated in Figure S19, similar to the baseline parameters.

## 4. Conclusions

Based on the above experiments, the performance of p-CuO/n-ZnO heterojunction pyroelectric photodetectors was optimized using magnetron sputtering technology and leveraging the pyroelectric photoelectric effect. The devices were systematically investigated for their photoresponse to light wavelengths of 365 nm and 405 nm under various optical power densities. The results demonstrate that integrating the pyroelectric photoelectric effect significantly enhances the device’s photocurrent and responsivity by more than an order of magnitude. Furthermore, the impact of different sputtering parameters of CuO on the device performance was studied. It was found that for p-CuO/n-ZnO heterojunction pyroelectric photodetectors, the optimal sputtering parameters of CuO are as follows: sputtering power of 120 W, sputtering duration of 15 min, and an oxygen–argon ratio of 1:3. This work not only provides a novel and effective approach to enhance the performance of light detectors using the pyroelectric photoelectric effect but also further explores the optimal sputtering parameters for CuO, offering insights for manufacturing high-performance pyroelectric photodetectors.

## Data Availability

The raw data supporting the conclusions of this article will be made available by the authors on request.

## Supplementary Materials

The following supporting information can be downloaded at: https://www.mdpi.com/article/10.3390/s24248197/s1. Figures S1 to S23 are included in the Supplementary Materials.
