# Supplementary material for: p-CuO/n-ZnO Heterojunction Pyro-Phototronic Photodetector Controlled by CuO Preparation Parameters
Source: Sensors (Basel). 2024 Dec 22;24(24):8197. doi: 10.3390/s24248197 (PMC11678976; doi:10.3390/s24248197)
Supplement: Supplementary file 1 [file sensors-24-08197-s001.zip › sensors-3315600-supplementary.pdf]

Supporting Material

p-CuO/n-ZnO heterojunction pyro-phototronic photodetector controlled by CuO preparation parameters

Zhen Zhang[1,2], Fangpei Li[1,2,*], Wenbo Peng[1,2,*], Quanzhe Zhu[3], Yongning He[1,2]
[1]School of Microelectronics, Xi'an Jiaotong University, Xi'an, Shaanxi, 710049, China
[2]The Key Lab of Micro-Nano Electronics and System Integration of Xi'an City, Xi'an, Shaanxi, 710049, China
[3]Shaanxi Advanced Semiconductor Technology Center Co., Ltd., Xi'an 710077, China
[*]Corresponding authors. E-mail: lifangpei@xjtu.edu.cn (Fangpei Li), wpeng33@mail.xjtu.edu.cn (Wenbo Peng)

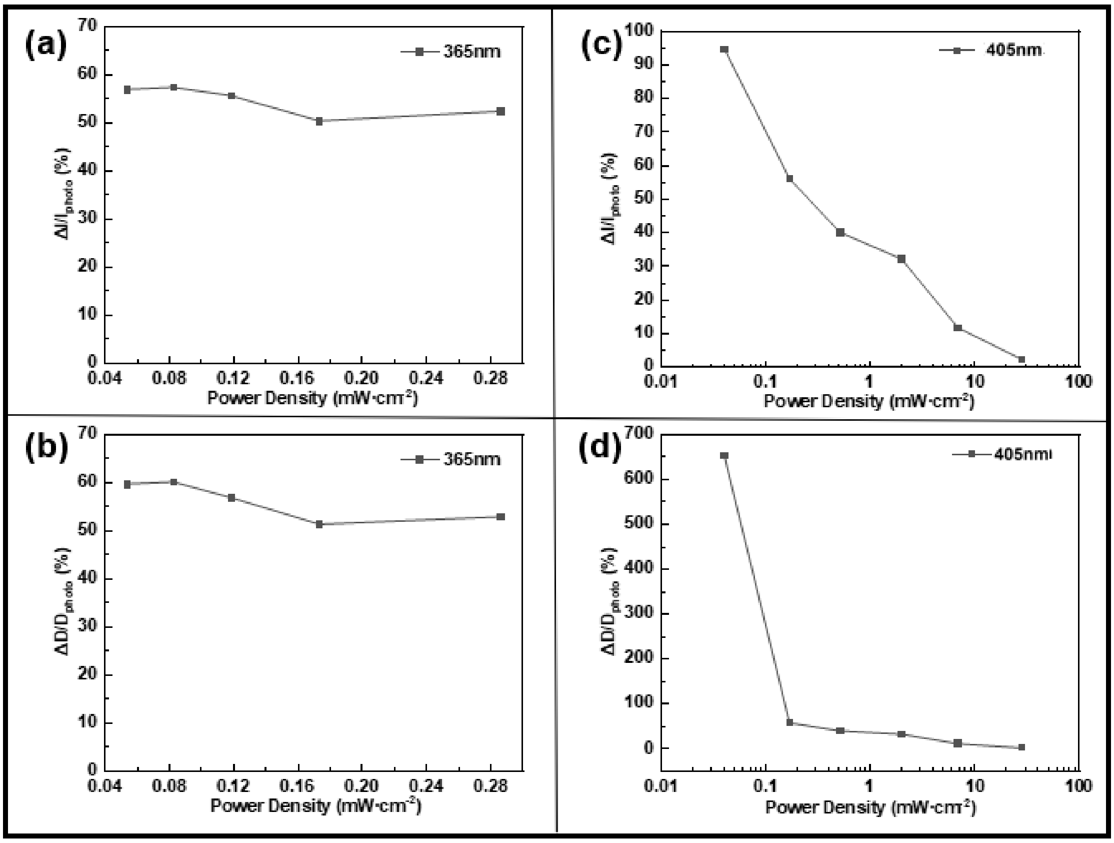

Figure S1. The maximum gain of the photocurrent (a) I_{photo} and the maximum gain of the detectivity (b) D_{photo} of the baseline parameter detector under different optical power densities at a 365 nm laser. Similarly, (c) depicts the maximum gain of I_{photo} and (d) illustrates the maximum gain of D_{photo} under various optical power densities at a 405 nm laser.

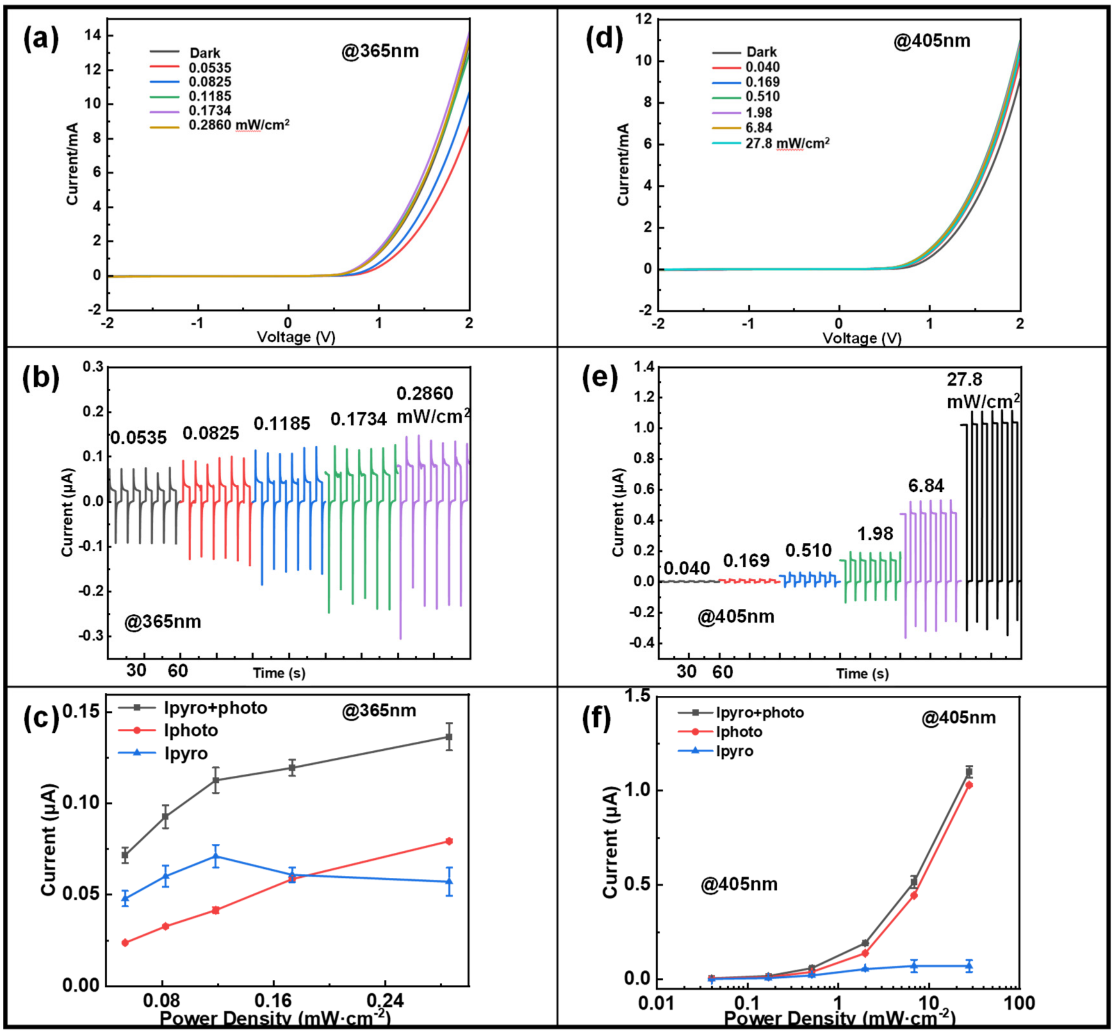

Figure S2. CuO sputtering power at 120 W for p-CuO/n-ZnO heterojunction pyroelectric photodetector at 365 nm laser: (a) I-V characteristics of photodetectors under dark and laser illumination of different power densities. (b) I-t transient responses of the device's (c) current components ($I_{pyro+photo}$, I_{photo}, and I_{pyro}) at each optical power density. CuO sputtering power at 120 W for p-CuO/n-ZnO heterojunction pyroelectric photodetector at a 405 nm laser: (d) I-V characteristics of photodetectors under dark and laser illumination of different power densities. (e) I-t transient responses of the device's (f) current components ($I_{pyro+photo}$, I_{photo}, and I_{pyro}) at each optical power density.

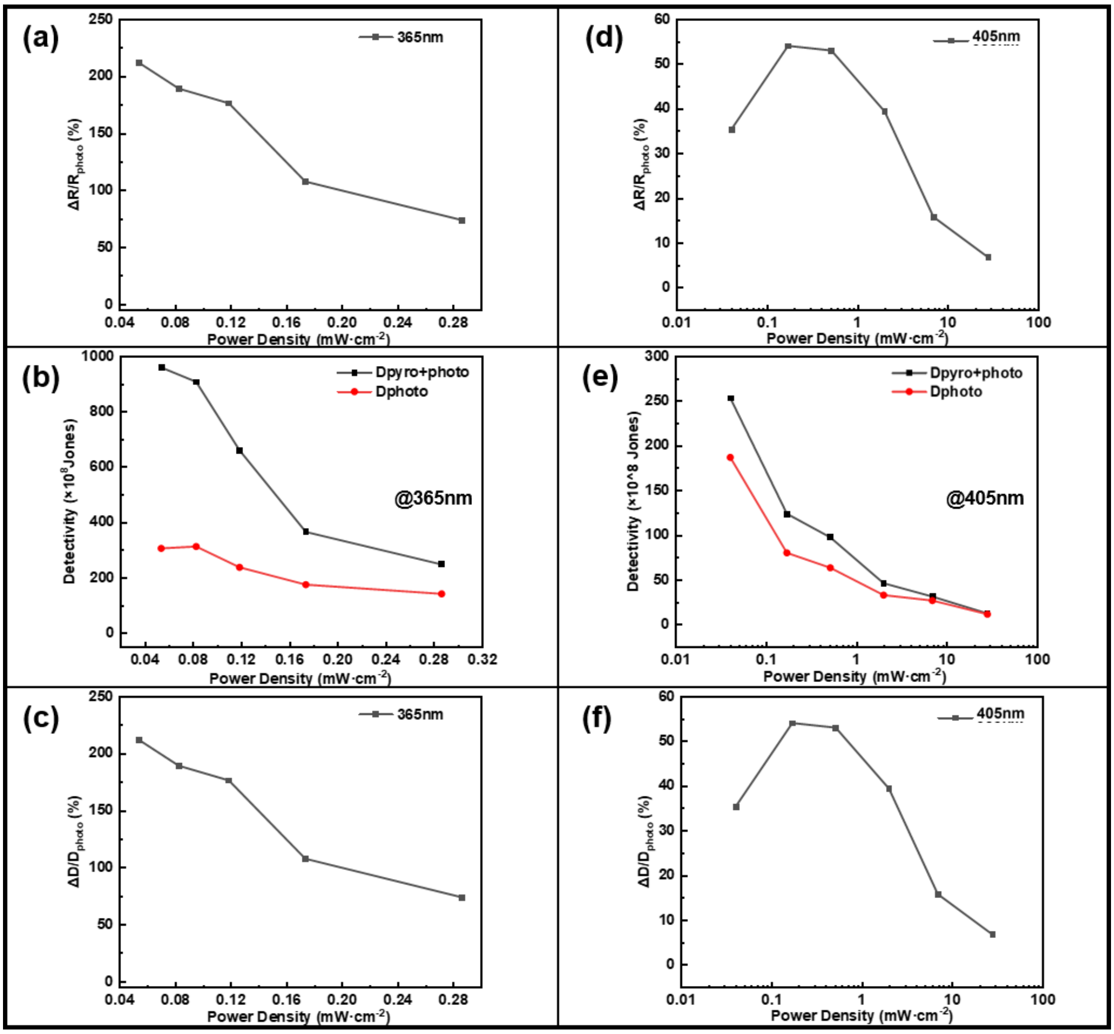

Figure S3. CuO sputtering power at 120 W for p-CuO/n-ZnO heterojunction pyroelectric photodetector at a 365 nm laser: (a) The maximum gain of responsivity $\Delta R/R_{photo}$ as a function of power density. (b) The detectivity as a function of power density. (c) The maximum gain of detectivity $\Delta D/D_{photo}$ as a function of power density. CuO sputtering power at 120 W for p-CuO/n-ZnO heterojunction pyroelectric photodetector at 405 nm laser: (d) The maximum gain of responsivity $\Delta R/R_{photo}$ as a function of power density. (e) The detectivity as a function of power density. (f) The maximum gain of detectivity $\Delta D/D_{photo}$ as a function of power density.

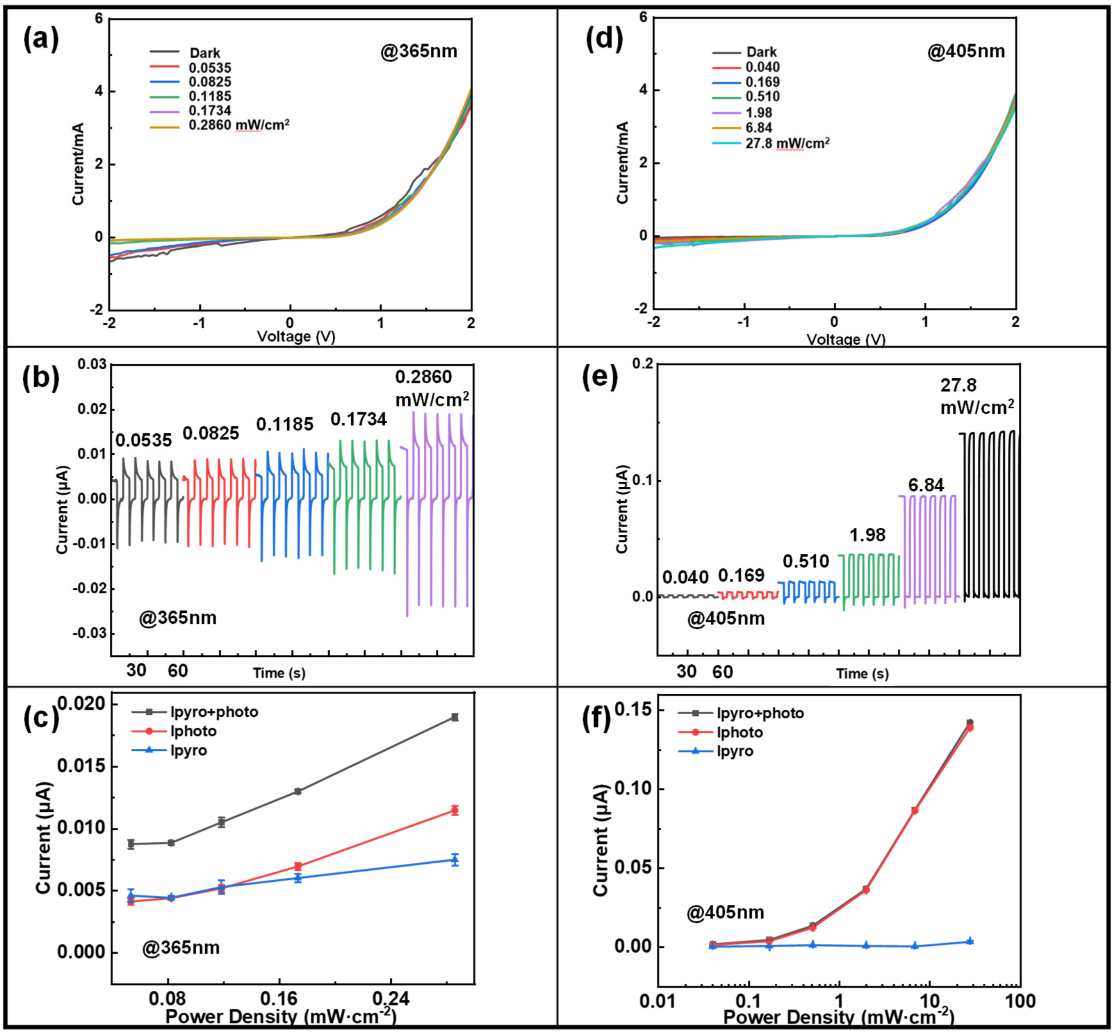

Figure S4. CuO sputtering power at 140 W for p-CuO/n-ZnO heterojunction pyroelectric photodetector at a 365 nm laser: (a) I-V characteristics of photodetectors under dark and laser illumination of different power densities. (b) I-t transient responses of the device's (c) current components ($I_{pyro+photo}$, I_{photo}, and I_{pyro}) at each optical power density. CuO sputtering power at 140 W for p-CuO/n-ZnO heterojunction pyroelectric photodetector at 405 nm laser: (d) I-V characteristics of photodetectors under dark and laser illumination of different power densities. (e) I-t transient responses of the device's (f) current components ($I_{pyro+photo}$, I_{photo}, and I_{pyro}) at each optical power density.

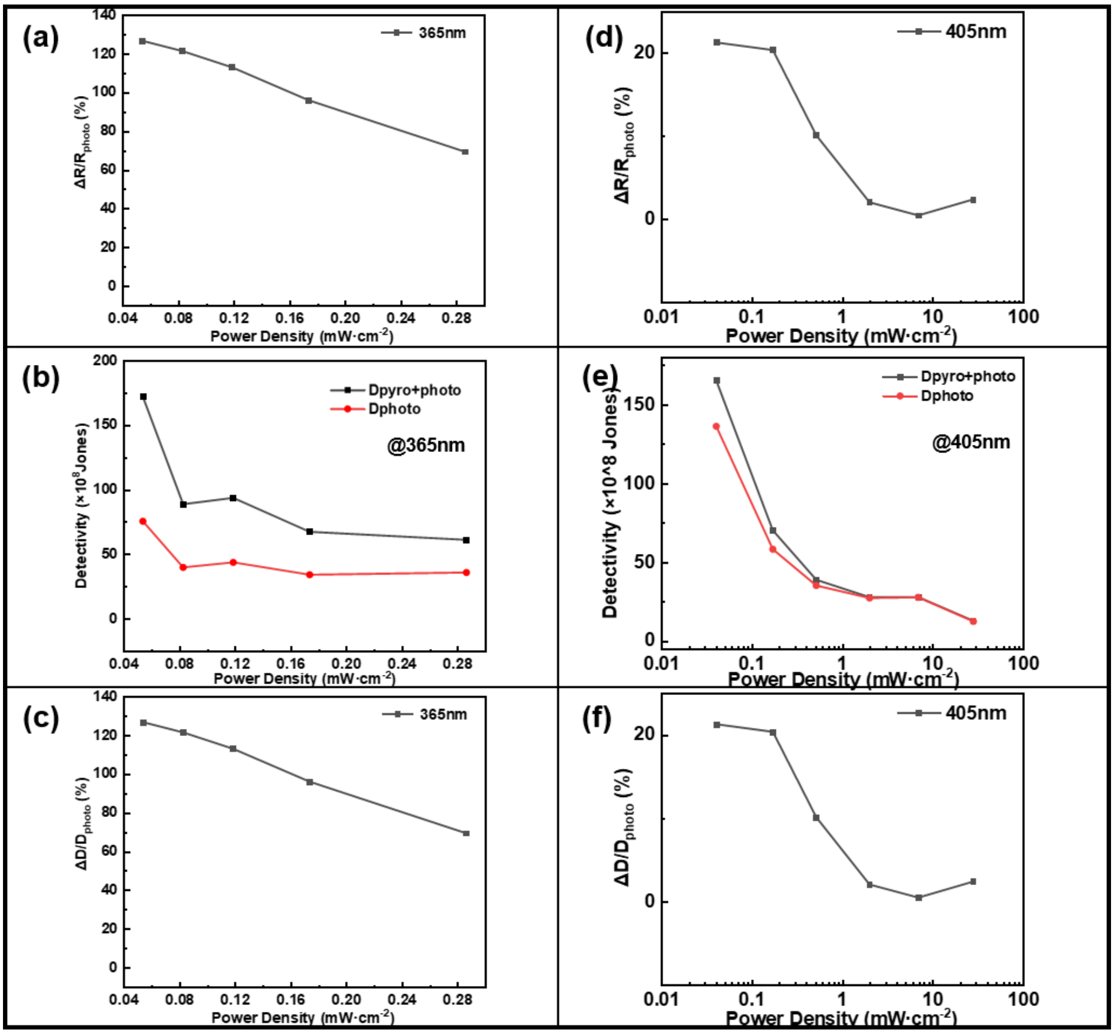

Figure S5. CuO sputtering power at 140 W for p-CuO/n-ZnO heterojunction pyroelectric photodetector at a 365 nm laser: (a) The maximum gain of responsivity $\Delta R/R_{photo}$ as a function of power density. (b) The detectivity as a function of power density. (c) The maximum gain of detectivity $\Delta D/D_{photo}$ as a function of power density. CuO sputtering power at 140 W for p-CuO/n-ZnO heterojunction pyroelectric photodetector at 405 nm laser: (d) The maximum gain of responsivity $\Delta R/R_{photo}$ as a function of power density. (e) The detectivity as a function of power density. (f) The maximum gain of detectivity $\Delta D/D_{photo}$ as a function of power density.

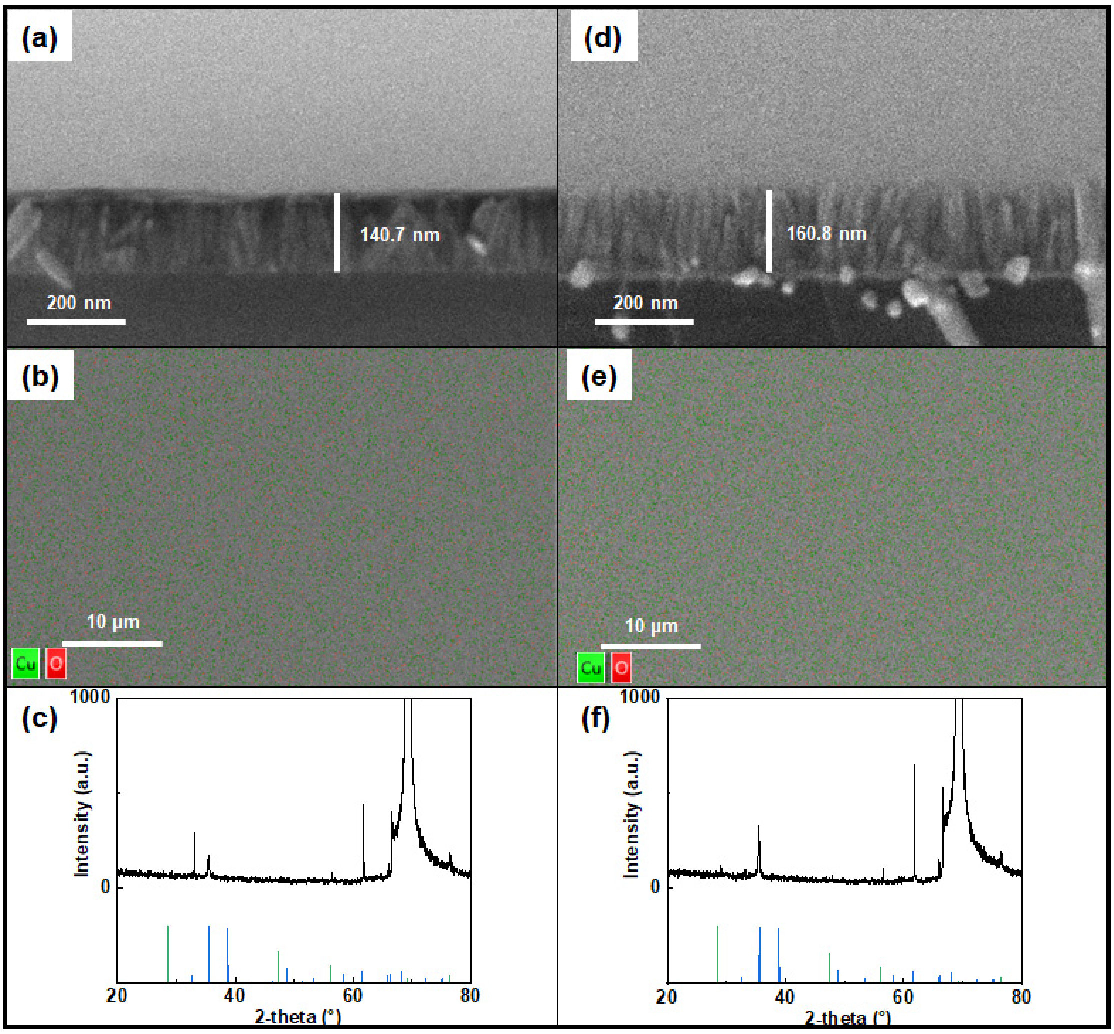

Figure S6. CuO sputtering power at 120 W for p-CuO/n-ZnO heterojunction pyroelectric photodetector: (a) Side-view SEM image. (b) EDS spectrum. (c) XRD pattern. CuO sputtering power at 140 W for p-CuO/n-ZnO heterojunction pyroelectric photodetector: (d) Side-view SEM image. (e) EDS spectrum. (f) XRD pattern.

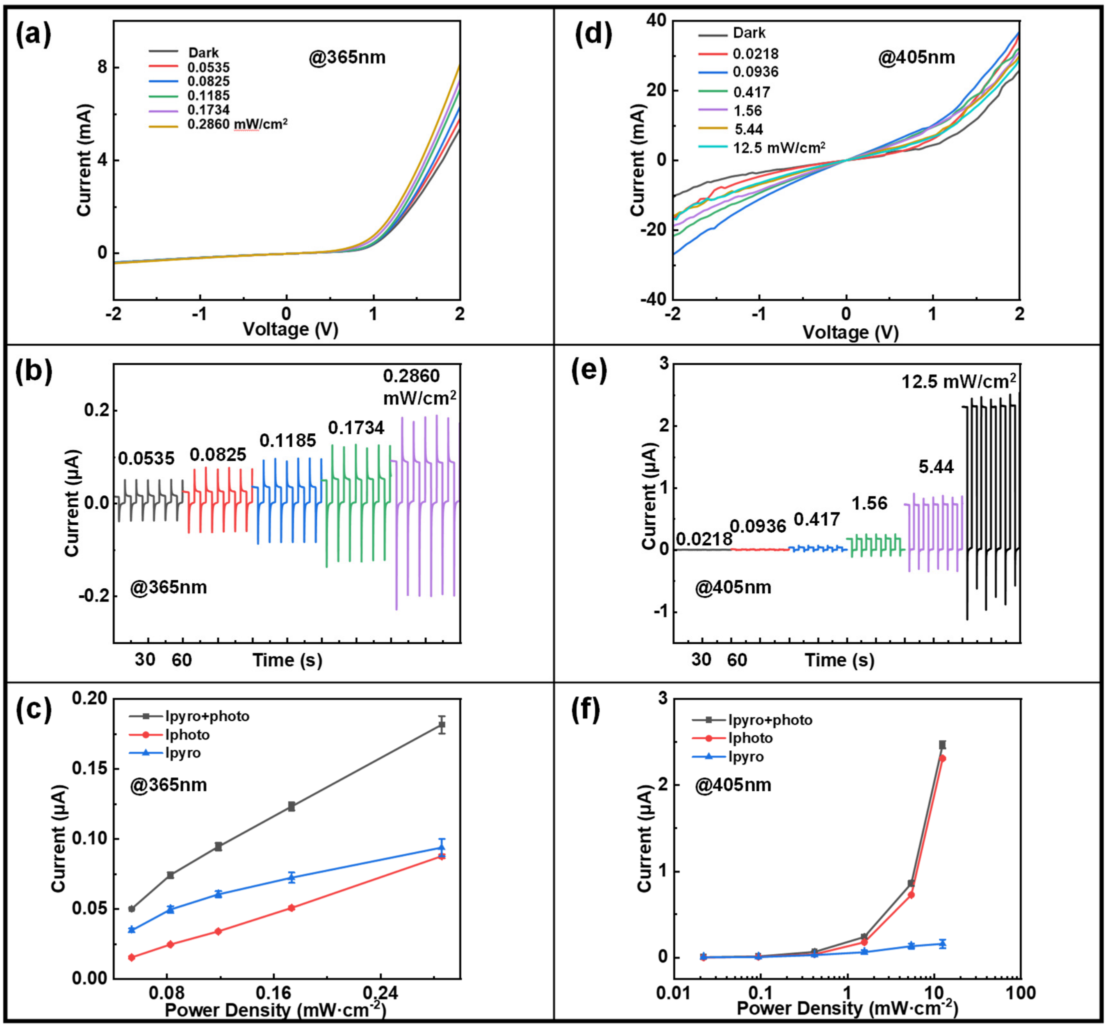

Figure S7. CuO sputtering time at 15 min for p-CuO/n-ZnO heterojunction pyroelectric photodetector at a 365 nm laser: (a) I-V characteristics of photodetectors under dark and laser illumination of different power densities. (b) I-t transient responses of the device's (c) current components ($I_{pyro+photo}$, I_{photo}, and I_{pyro}) at each optical power density. CuO sputtering time at 15 min for p-CuO/n-ZnO heterojunction pyroelectric photodetector at 405 nm laser: (d) I-V characteristics of photodetectors under dark and laser illumination of different power densities. (e) I-t transient responses of the device's (f) current components ($I_{pyro+photo}$, I_{photo}, and I_{pyro}) at each optical power density.

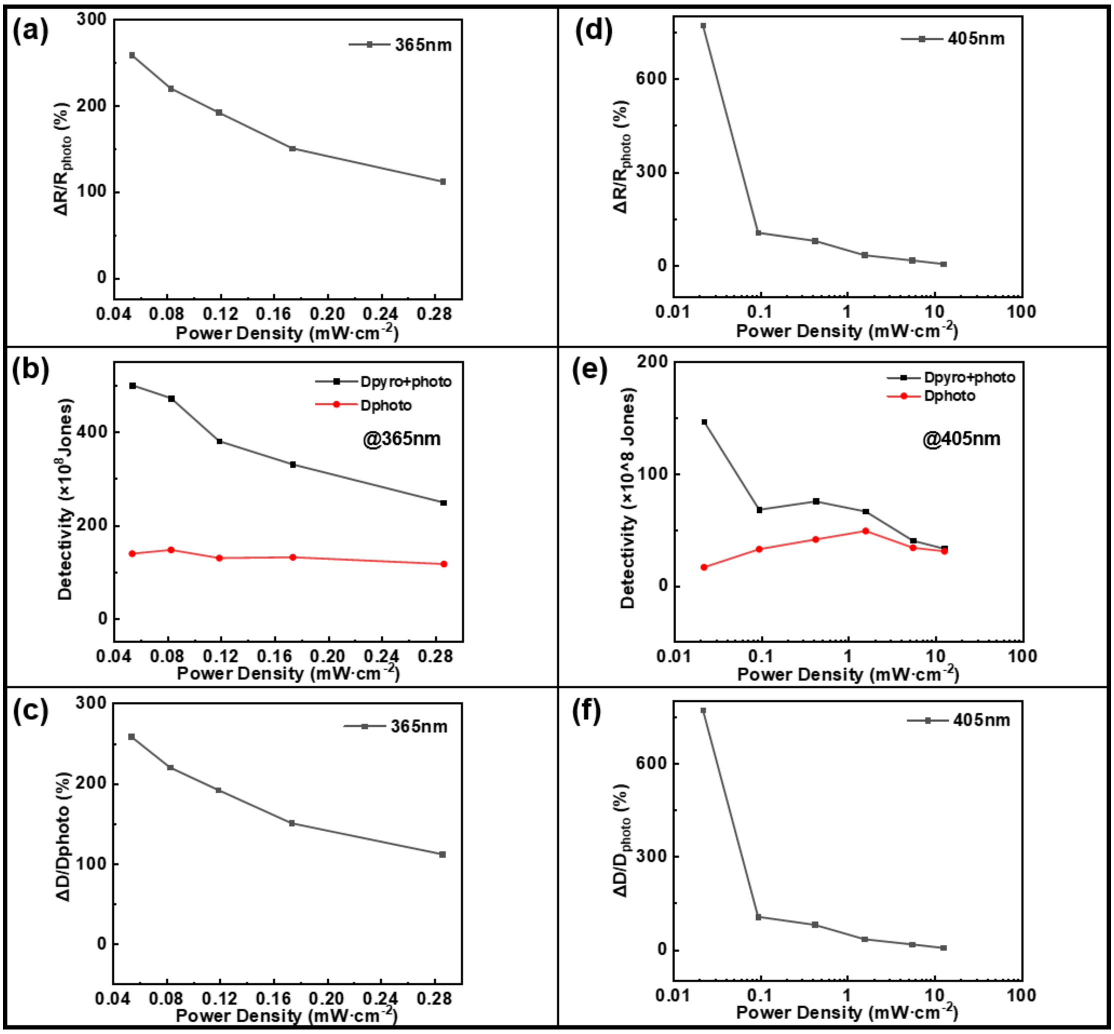

Figure S8. CuO sputtering time at 15 min for p-CuO/n-ZnO heterojunction pyroelectric photodetector at a 365 nm laser: (a) The maximum gain of responsivity $\Delta R/R_{photo}$ as a function of power density. (b) The detectivity as a function of power density. (c) The maximum gain of detectivity $\Delta D/D_{photo}$ as a function of power density. CuO sputtering time at 15 min for p-CuO/n-ZnO heterojunction pyroelectric photodetector at a 405 nm laser: (d) The maximum gain of responsivity $\Delta R/R_{photo}$ as a function of power density. (e) The detectivity as a function of power density. (f) The maximum gain of detectivity $\Delta D/D_{photo}$ as a function of power density.

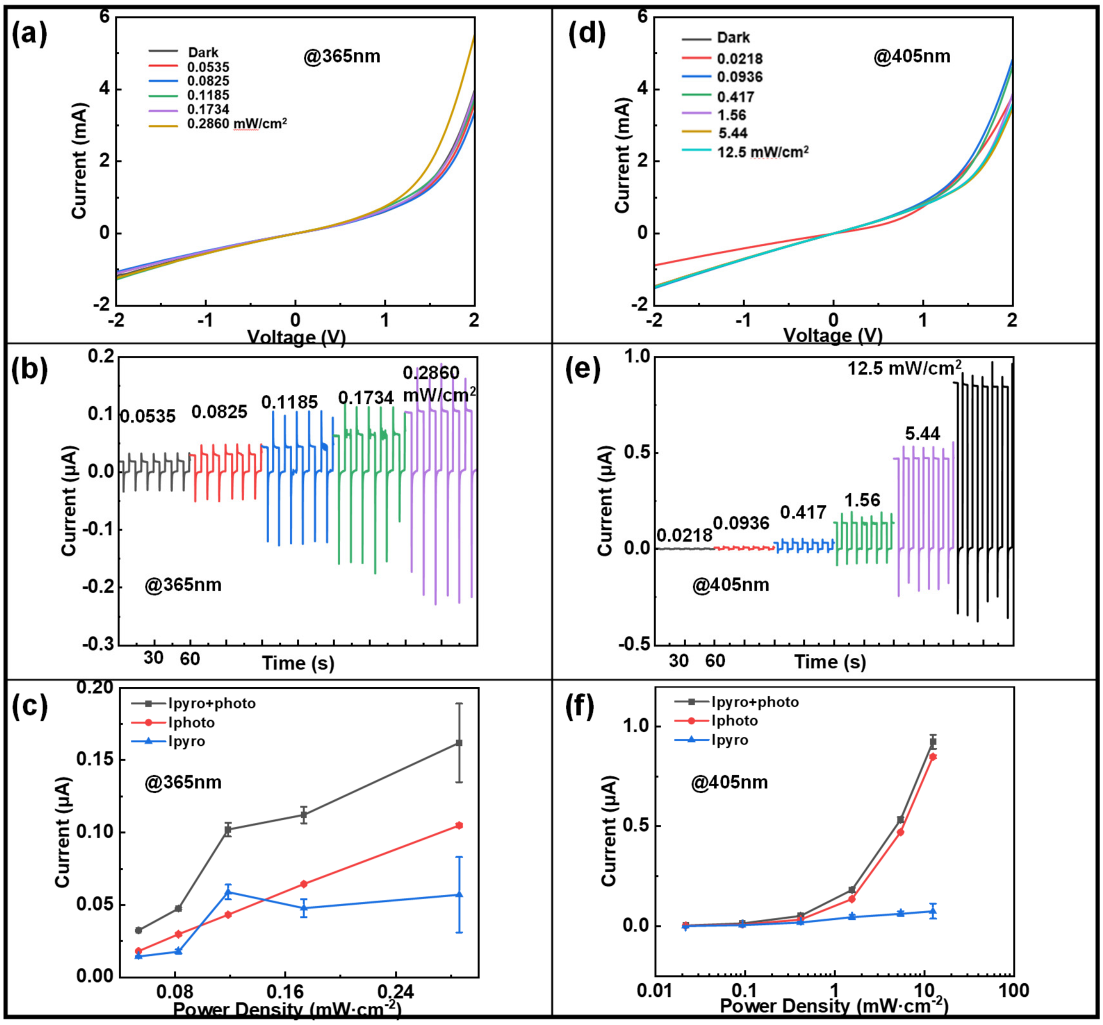

Figure S9. CuO sputtering time at 45 min for p-CuO/n-ZnO heterojunction pyroelectric photodetector at a 365 nm laser: (a) I-V characteristics of photodetectors under dark and laser illumination of different power densities. (b) I-t transient responses of the device's (c) current components ($I_{pyro+photo}$, I_{photo}, and I_{pyro}) at each optical power density. CuO sputtering time at 45 min for p-CuO/n-ZnO heterojunction pyroelectric photodetector at a 405 nm laser: (d) I-V characteristics of photodetectors under dark and laser illumination of different power densities. (e) I-t transient responses of the device's (f) current components ($I_{pyro+photo}$, I_{photo}, and I_{pyro}) at each optical power density.

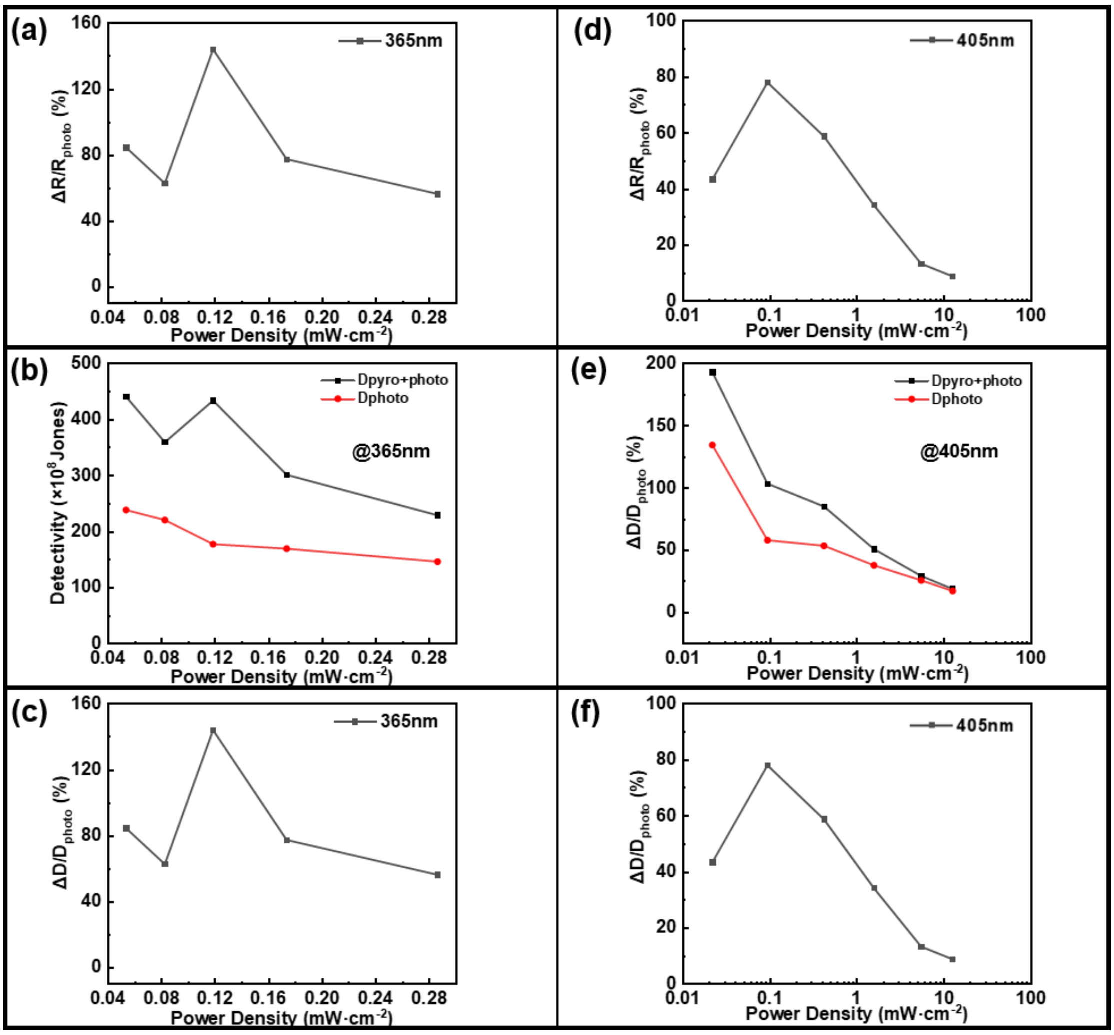

Figure S10. CuO sputtering time at 45 min for p-CuO/n-ZnO heterojunction pyroelectric photodetector at a 365 nm laser: (a) The maximum gain of responsivity $\Delta R/R_{photo}$ as a function of power density. (b) The detectivity as a function of power density. (c) The maximum gain of detectivity $\Delta D/D_{photo}$ as a function of power density. CuO sputtering time at 45 min for p-CuO/n-ZnO heterojunction pyroelectric photodetector at a 405 nm laser: (d) The maximum gain of responsivity $\Delta R/R_{photo}$ as a function of power density. (e) The detectivity as a function of power density. (f) The maximum gain of detectivity $\Delta D/D_{photo}$ as a function of power density.

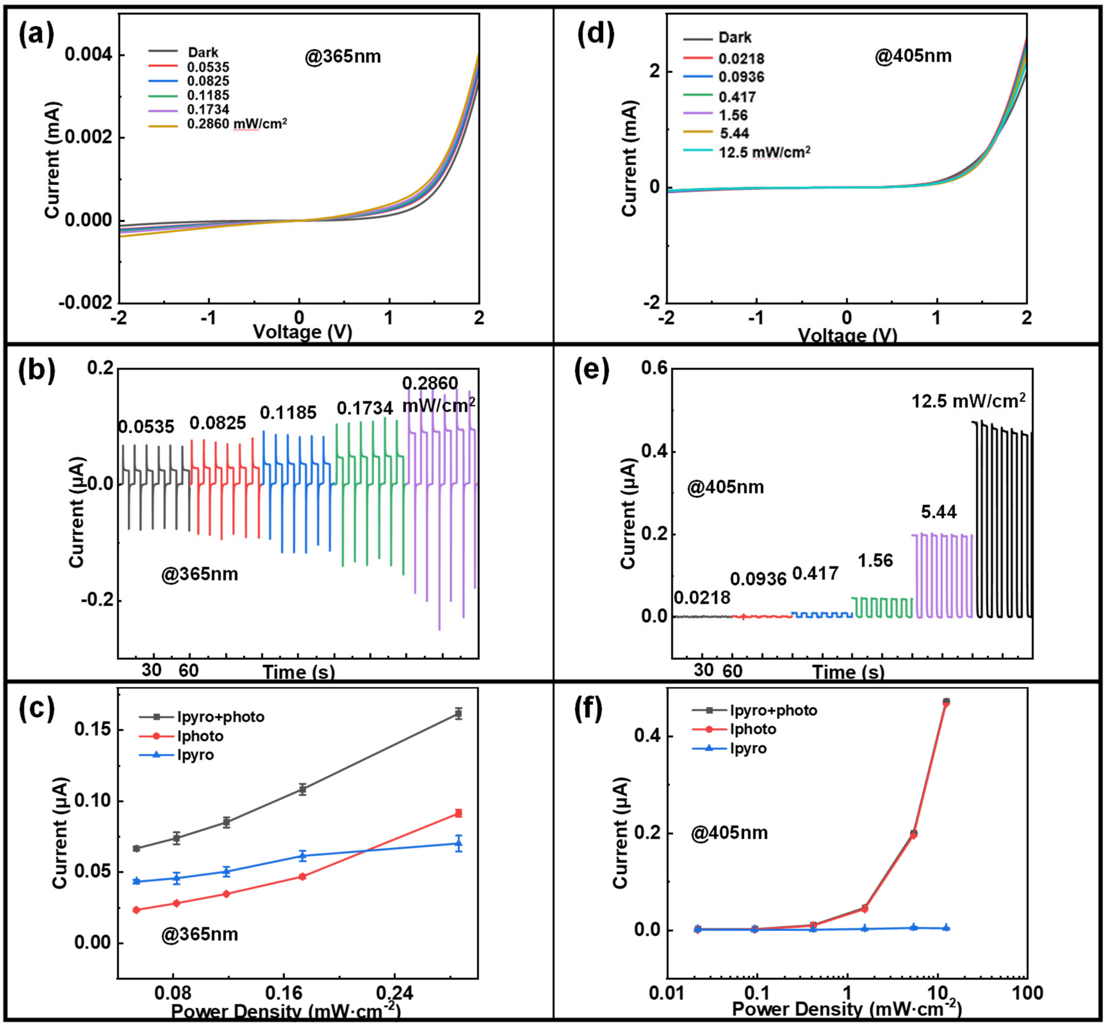

Figure S11. CuO sputtering time at 60 min for p-CuO/n-ZnO heterojunction pyroelectric photodetector at a 365 nm laser: (a) I-V characteristics of photodetectors under dark and laser illumination of different power densities. (b) I-t transient responses of the device's (c) current components ($I_{pyro+photo}$, I_{photo}, and I_{pyro}) at each optical power density. CuO sputtering time at 60 min for p-CuO/n-ZnO heterojunction pyroelectric photodetector at a 405 nm laser: (d) I-V characteristics of photodetectors under dark and laser illumination of different power densities. (e) I-t transient responses of the device's (f) current components ($I_{pyro+photo}$, I_{photo}, and I_{pyro}) at each optical power density.

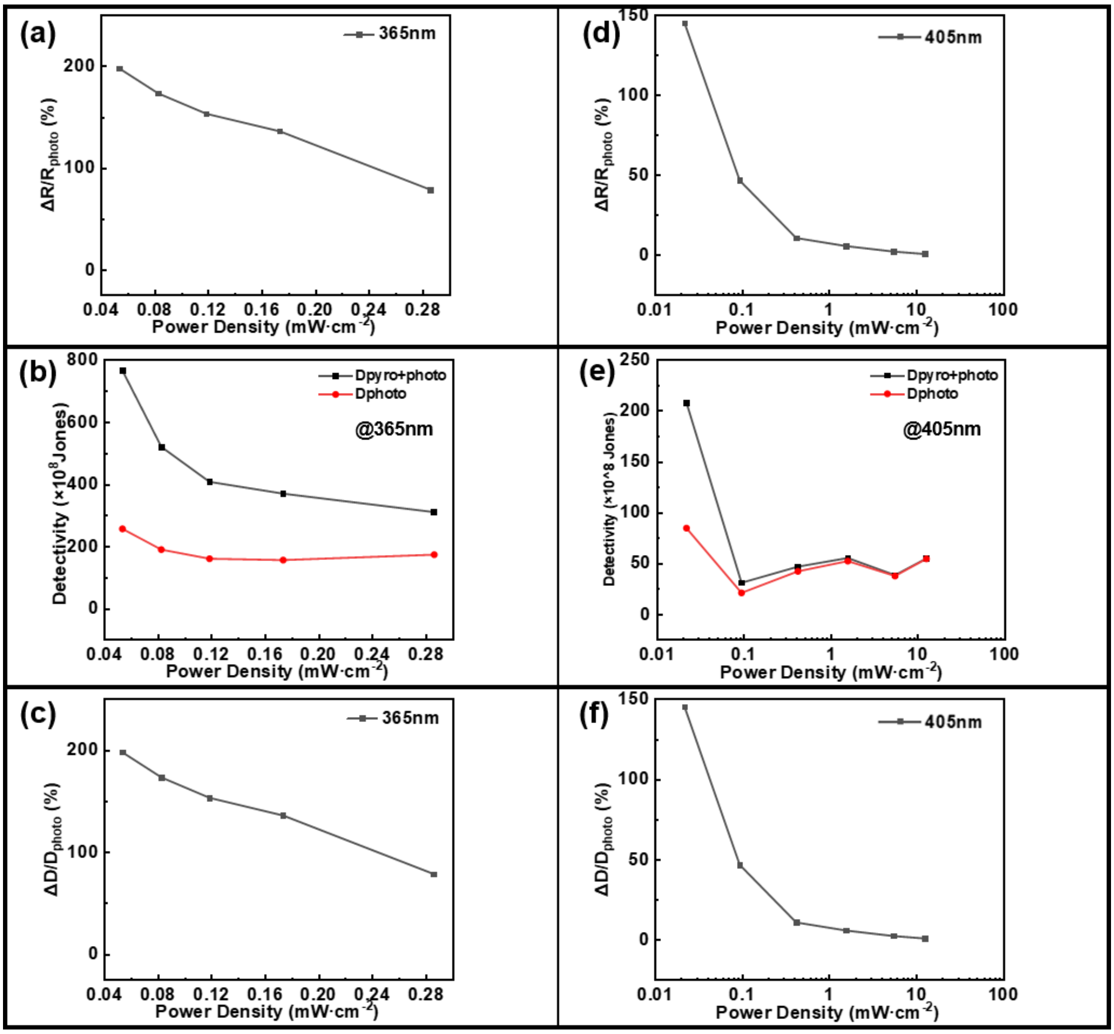

Figure S12. CuO sputtering time at 60 min for p-CuO/n-ZnO heterojunction pyroelectric photodetector at a 365 nm laser: (a) The maximum gain of responsivity $\Delta R/R_{photo}$ as a function of power density. (b) The detectivity as a function of power density. (c) The maximum gain of detectivity $\Delta D/D_{photo}$ as a function of power density. CuO sputtering time at 60 min for p-CuO/n-ZnO heterojunction pyroelectric photodetector at a 405 nm laser: (d) The maximum gain of responsivity $\Delta R/R_{photo}$ as a function of power density. (e) The detectivity as a function of power density. (f) The maximum gain of detectivity $\Delta D/D_{photo}$ as a function of power density.

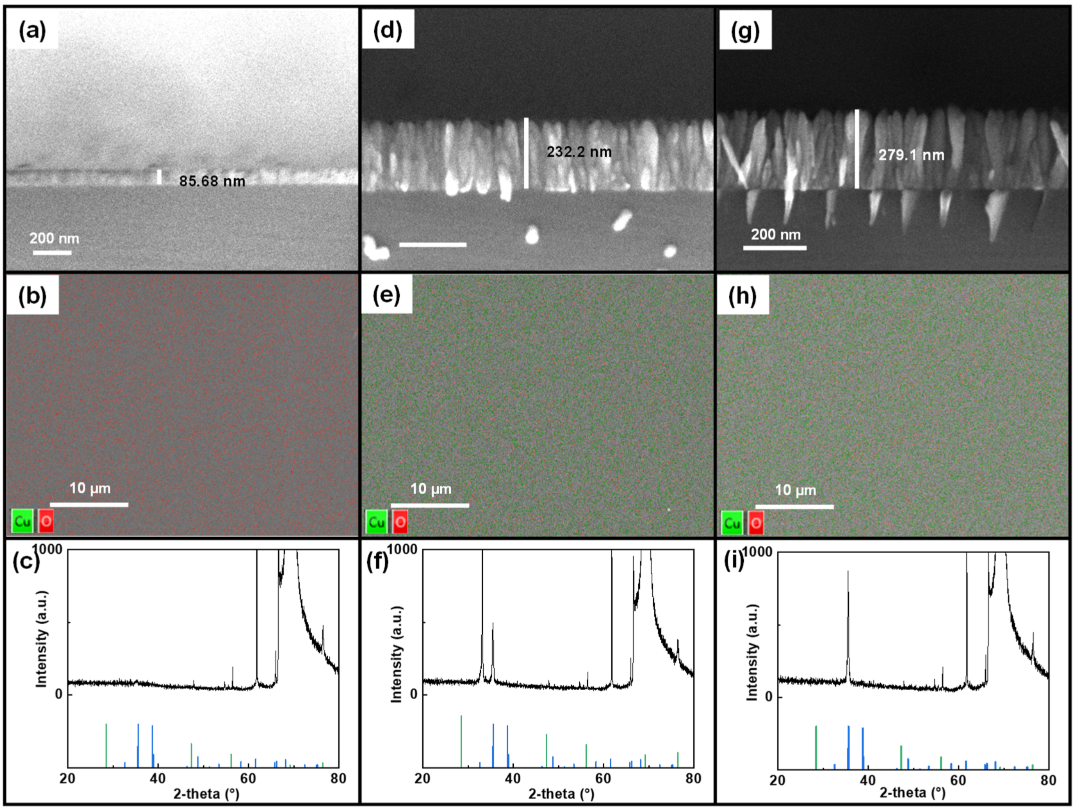

Figure S13. CuO sputtering time at 15 min for p-CuO/n-ZnO heterojunction pyroelectric photodetector: (a) Side-view SEM image. (b) EDS spectrum. (c) XRD pattern. CuO sputtering time at 45 min for p-CuO/n-ZnO heterojunction pyroelectric photodetector: (d) Side-view SEM image. (e) EDS spectrum. (f) XRD pattern. CuO sputtering time at 60 min for p-CuO/n-ZnO heterojunction pyroelectric photodetector: (g) Side-view SEM image. (h) EDS spectrum. (i) XRD pattern.

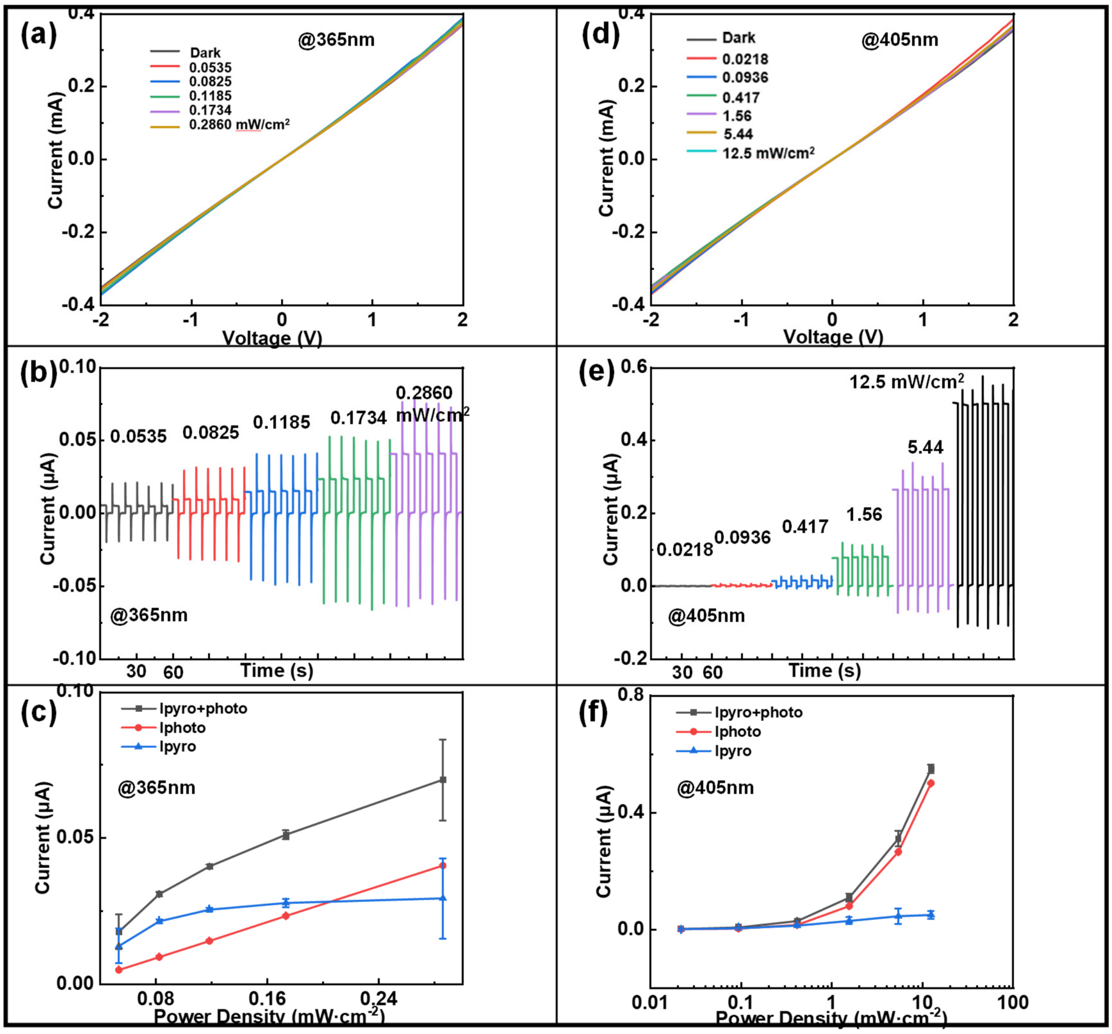

Figure S14. CuO sputtering oxygen–argon ratio for pure argon for p-CuO/n-ZnO heterojunction pyroelectric photodetector at a 365 nm laser: (a) I-V characteristics of photodetectors under dark and laser illumination of different power densities. (b) I-t transient responses of the device's (c) current components ($I_{pyro+photo}$, I_{photo}, and I_{pyro}) at each optical power density. CuO sputtering oxygen–argon ratio for pure argon for p-CuO/n-ZnO heterojunction pyroelectric photodetector at a 405 nm laser: (d) I-V characteristics of photodetectors under dark and laser illumination of different power densities. (e) I-t transient responses of the device's (f) current components ($I_{pyro+photo}$, I_{photo}, and I_{pyro}) at each optical power density.

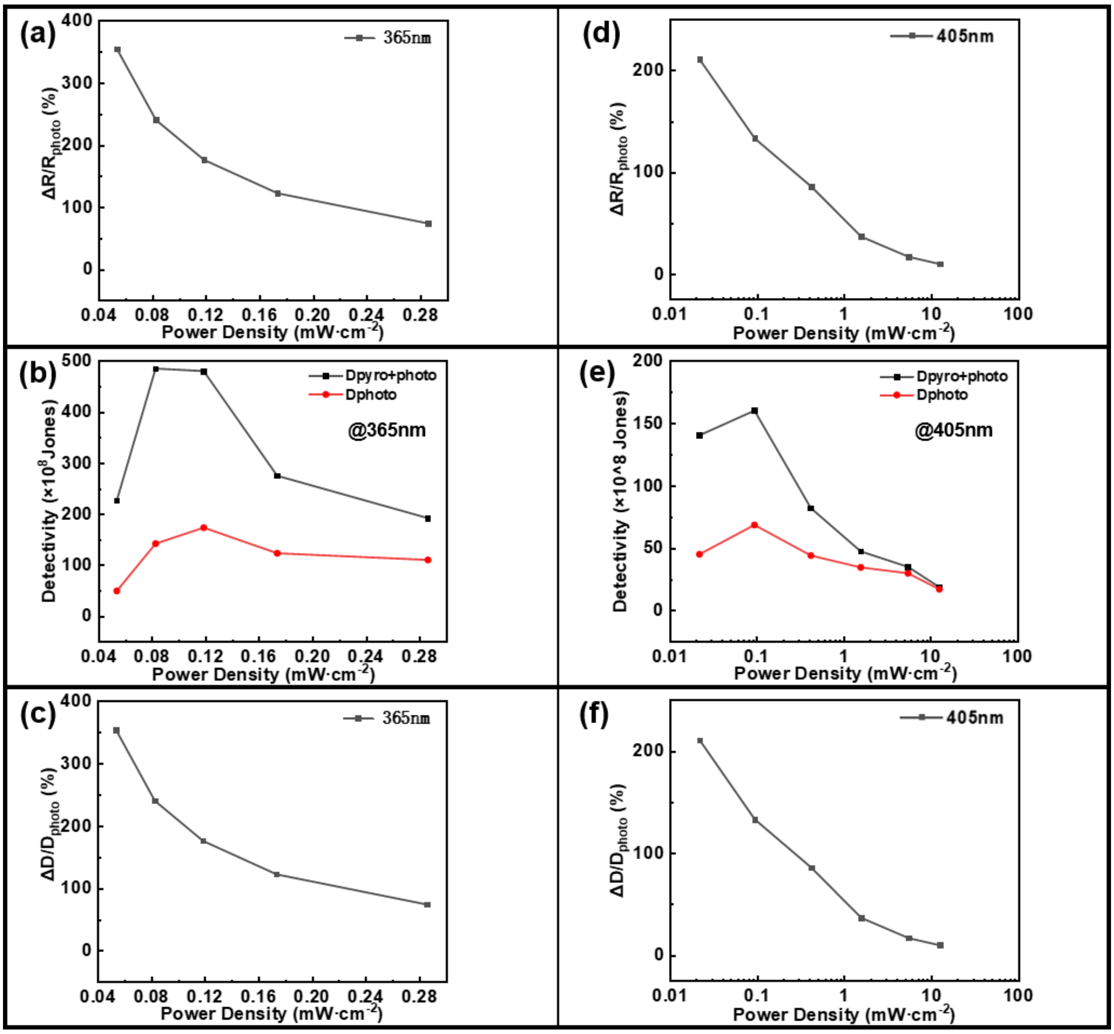

Figure S15. CuO sputtering oxygen–argon ratio for pure argon for p-CuO/n-ZnO heterojunction pyroelectric photodetector at a 365 nm laser: (a) The maximum gain of responsivity $\Delta R/R_{photo}$ as a function of power density. (b) The detectivity as a function of power density. (c) The maximum gain of detectivity $\Delta D/D_{photo}$ as a function of power density. CuO sputtering oxygen–argon ratio for pure argon for p-CuO/n-ZnO heterojunction pyroelectric photodetector at a 405 nm laser: (d) The maximum gain of responsivity $\Delta R/R_{photo}$ as a function of power density. (e) The detectivity as a function of power density. (f) The maximum gain of detectivity $\Delta D/D_{photo}$ as a function of power density.

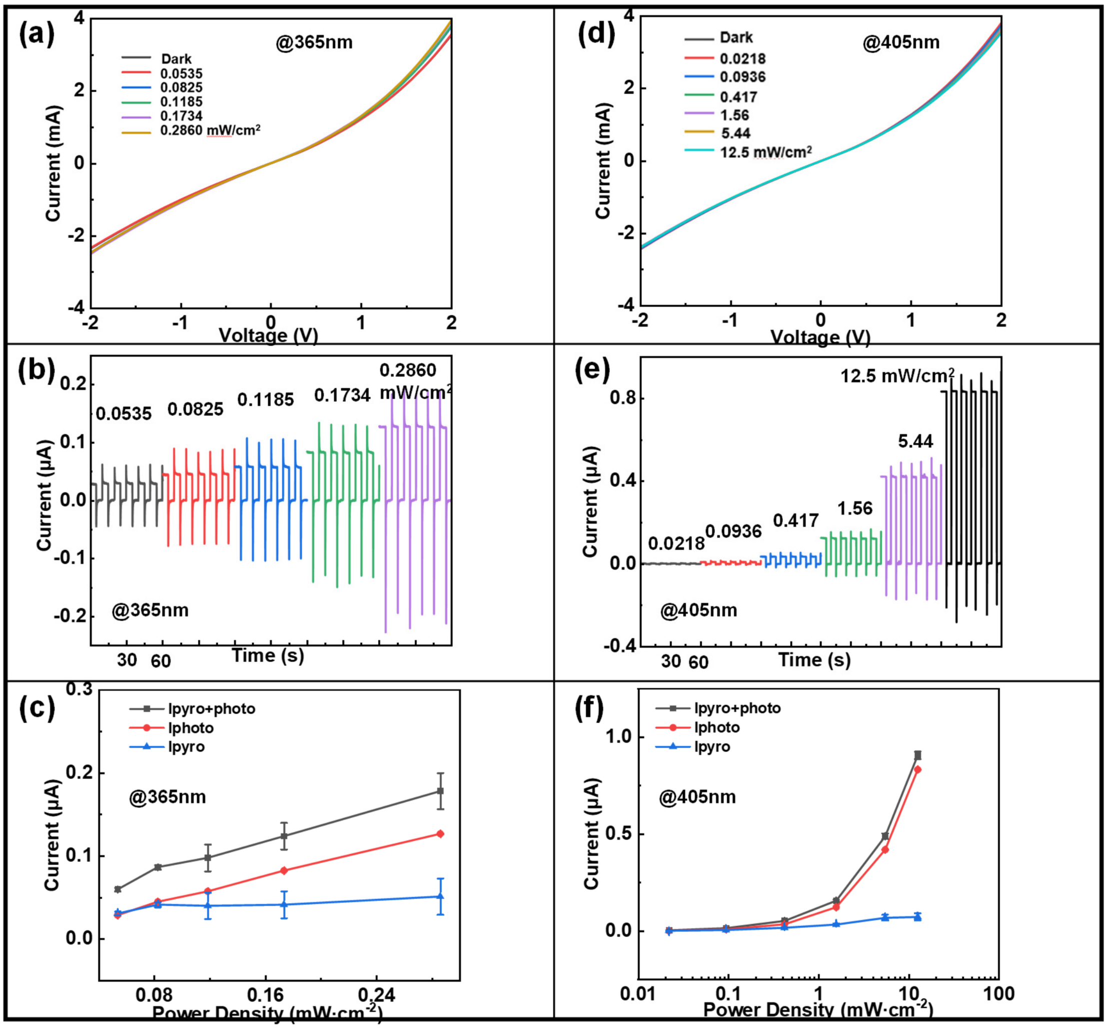

Figure S16. CuO sputtering oxygen–argon ratio for oxygen–argon ratio 1:1 for p-CuO/n-ZnO heterojunction pyroelectric photodetector at a 365 nm laser: (a) I-V characteristics of photodetectors under dark and laser illumination of different power densities. (b) I-t transient responses of the device's (c) current components ($I_{pyro+photo}$, I_{photo}, and I_{pyro}) at each optical power density. CuO sputtering oxygen–argon ratio for oxygen–argon ratio 1:1 for p-CuO/n-ZnO heterojunction pyroelectric photodetector at a 405 nm laser: (d) I-V characteristics of photodetectors under dark and laser illumination of different power densities. (e) I-t transient responses of the device's (f) current components ($I_{pyro+photo}$, I_{photo}, and I_{pyro}) at each optical power density.

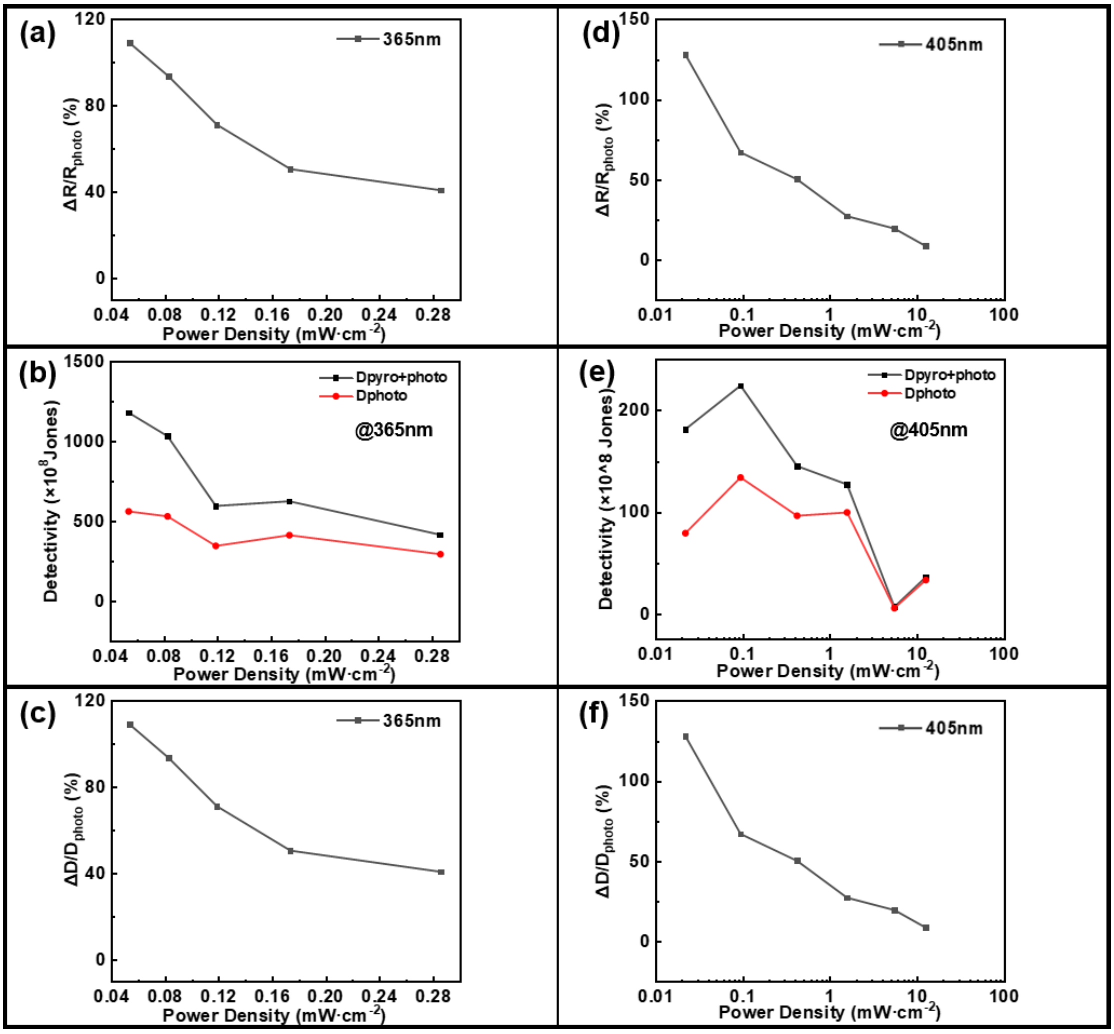

Figure S17. CuO sputtering oxygen–argon ratio for oxygen–argon ratio 1:1 argon for p-CuO/n-ZnO heterojunction pyroelectric photodetector at a 365 nm laser: (a) The maximum gain of responsivity $\Delta R/R_{photo}$ as a function of power density. (b) The detectivity as a function of power density. (c) The maximum gain of detectivity $\Delta D/D_{photo}$ as a function of power density. CuO sputtering oxygen–argon ratio for oxygen–argon ratio 1:1 argon for p-CuO/n-ZnO heterojunction pyroelectric photodetector at a 405 nm laser: (d) The maximum gain of responsivity $\Delta R/R_{photo}$ as a function of power density. (e) The detectivity as a function of power density. (f) The maximum gain of detectivity $\Delta D/D_{photo}$ as a function of power density.

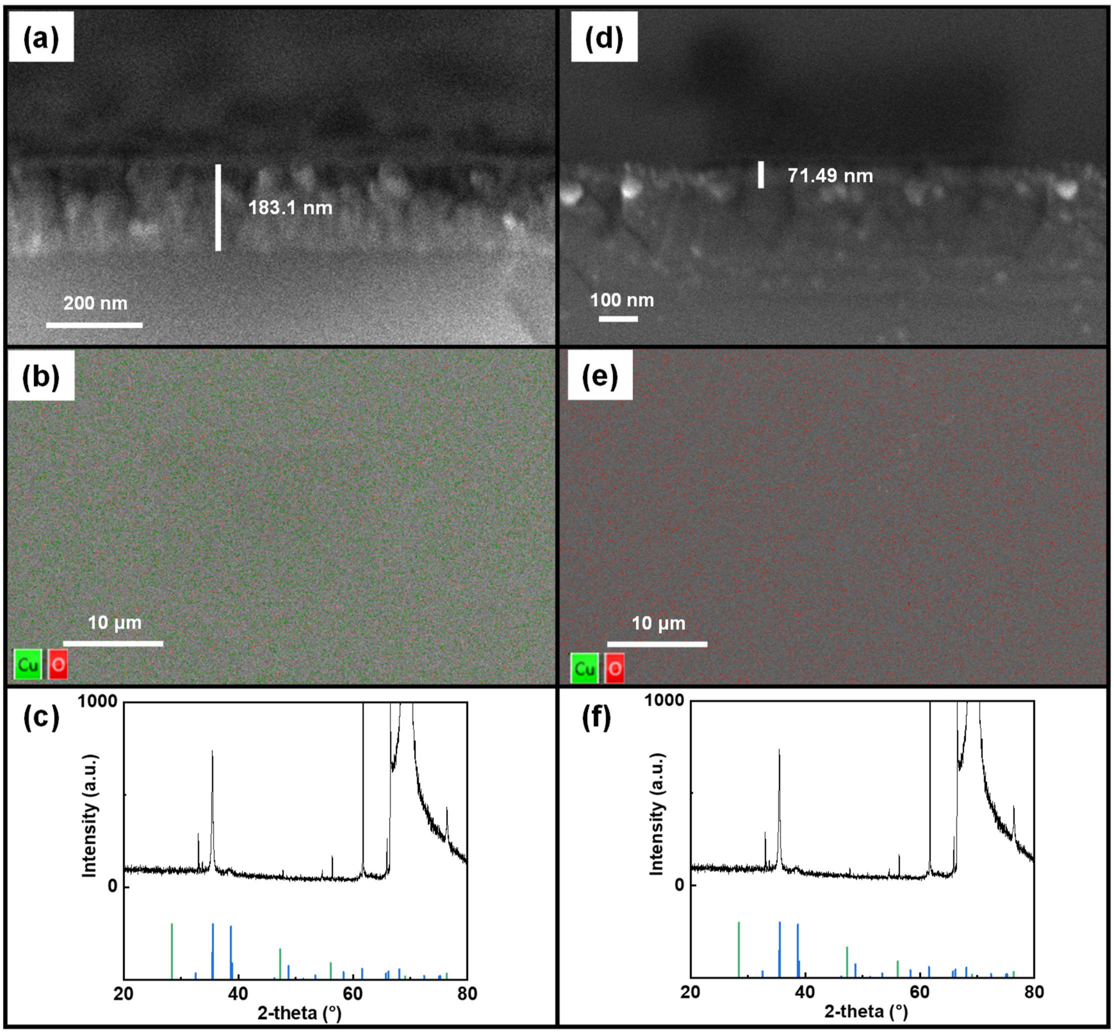

Figure S18. CuO sputtering oxygen–argon ratio for pure for p-CuO/n-ZnO heterojunction pyroelectric photodetector: (a) Side-view SEM image. (b) EDS spectrum. (c) XRD pattern. CuO sputtering oxygen–argon ratio for oxygen–argon ratio 1:1 for p-CuO/n-ZnO heterojunction pyroelectric photodetector: (d) Side-view SEM image. (e) EDS spectrum. (f) XRD pattern.

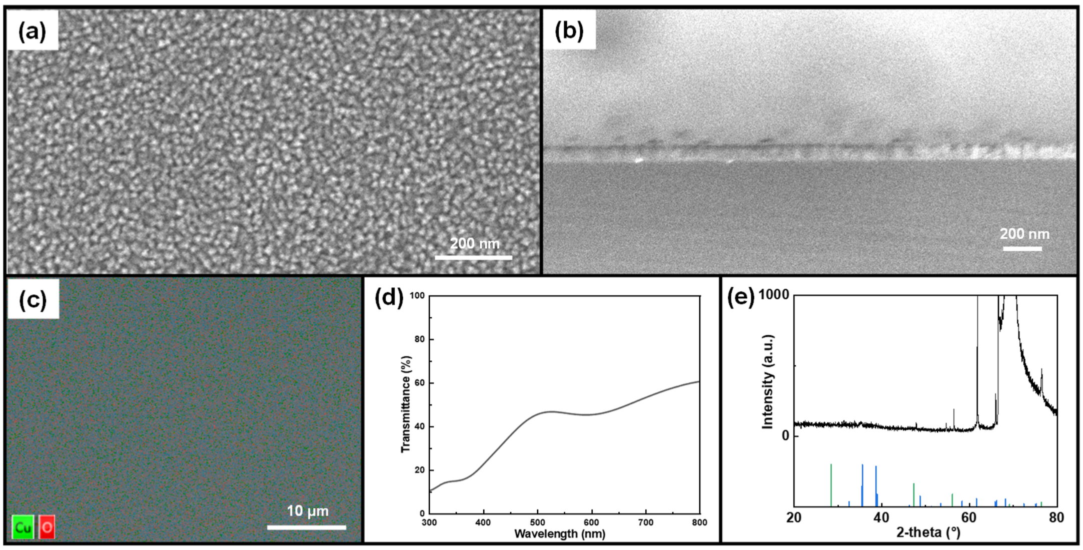

Figure S19. (a) Top-view SEM image of the device with optimal parameters. (b) Side-view SEM image of the device with optimal parameters. (c) EDS spectrum of CuO with optimal parameters. (d) Transmission spectrum of CuO with optimal parameters. (e) XRD pattern of CuO with optimal parameters.

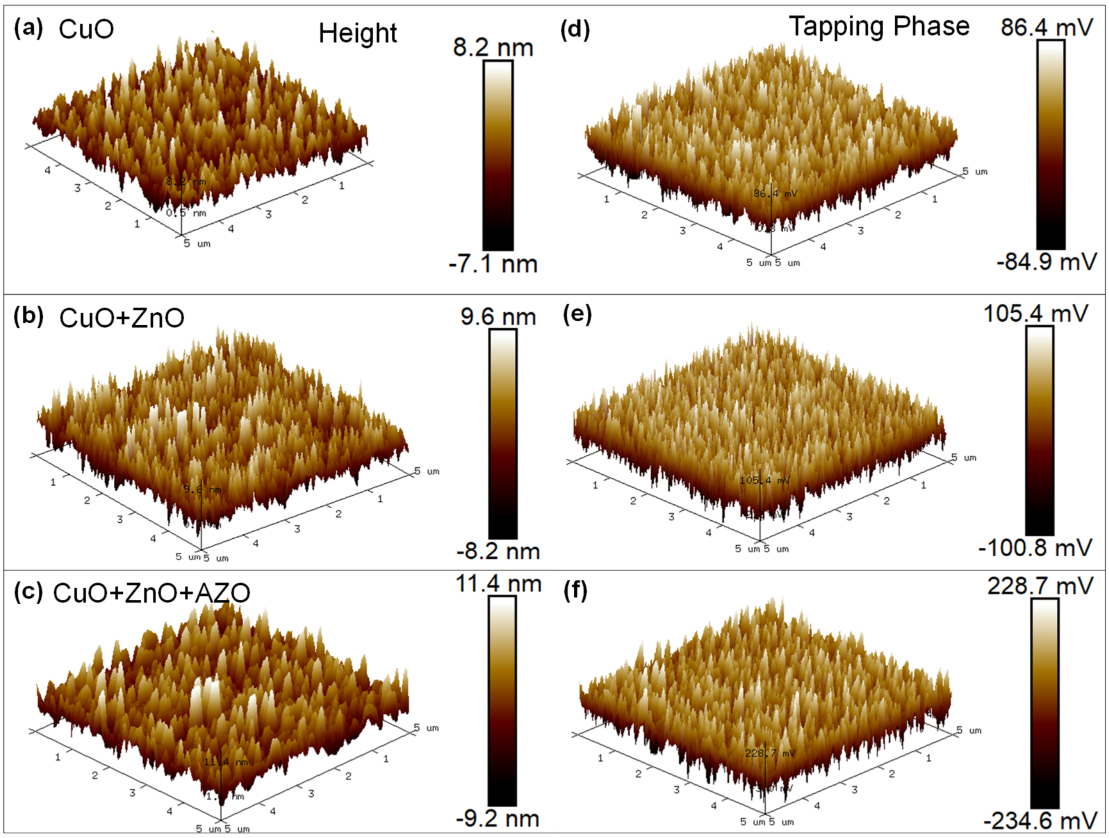

Figure S20. Surface height of films: (a) CuO; (b) CuO-ZnO; and (c) CuO-ZnO21. Tapping phase of films: (a) CuO; (b) CuO-ZnO; and (c) CuO-ZnO-AZO.

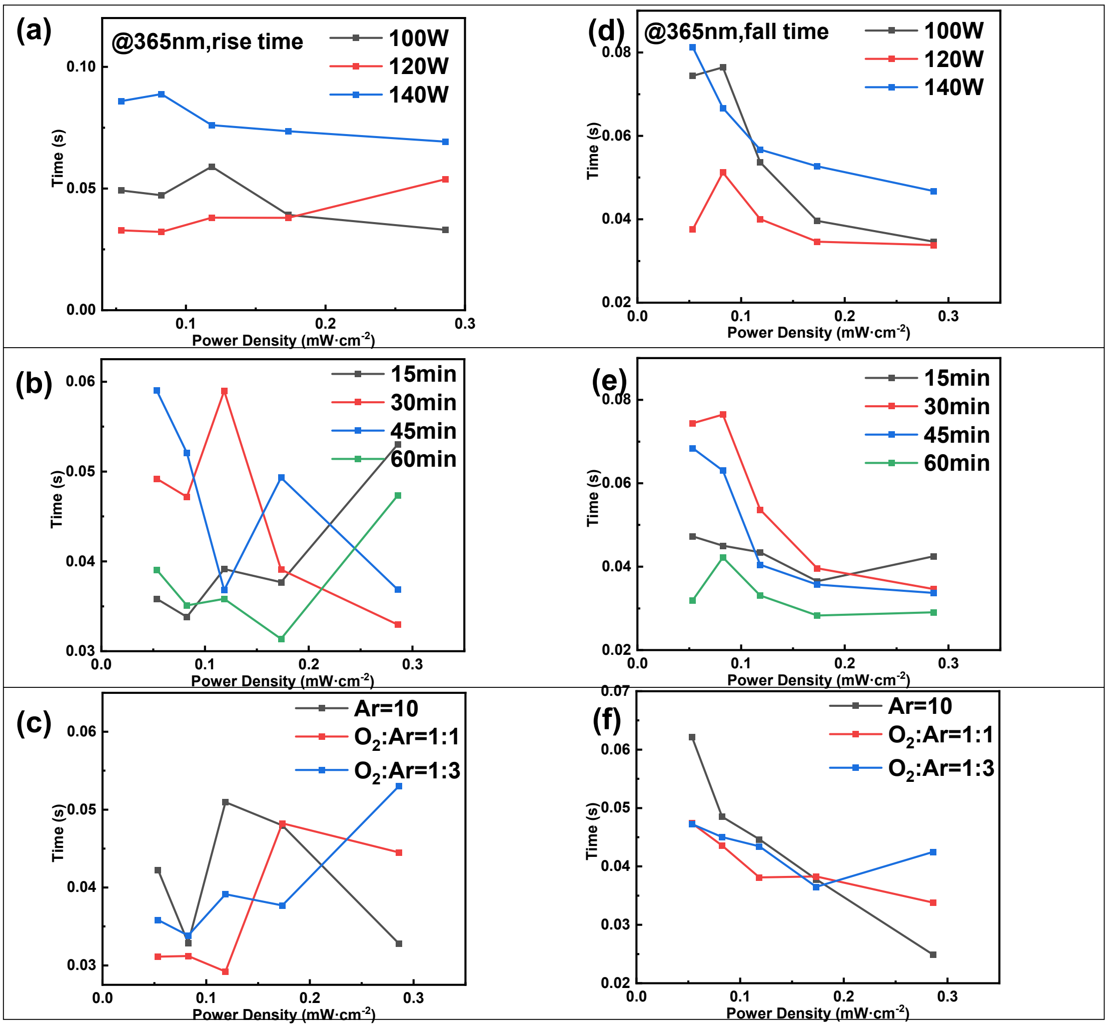

Figure S21. Device response rise time under a 365 nm illumination under different sputtering parameters: (a) sputtering time; (b) sputtering time; and (c) oxygen–argon ratio. Device response fall time under a 365 nm illumination under different sputtering parameters: (a) sputtering time; (b) sputtering time; and (c) oxygen–argon ratio.

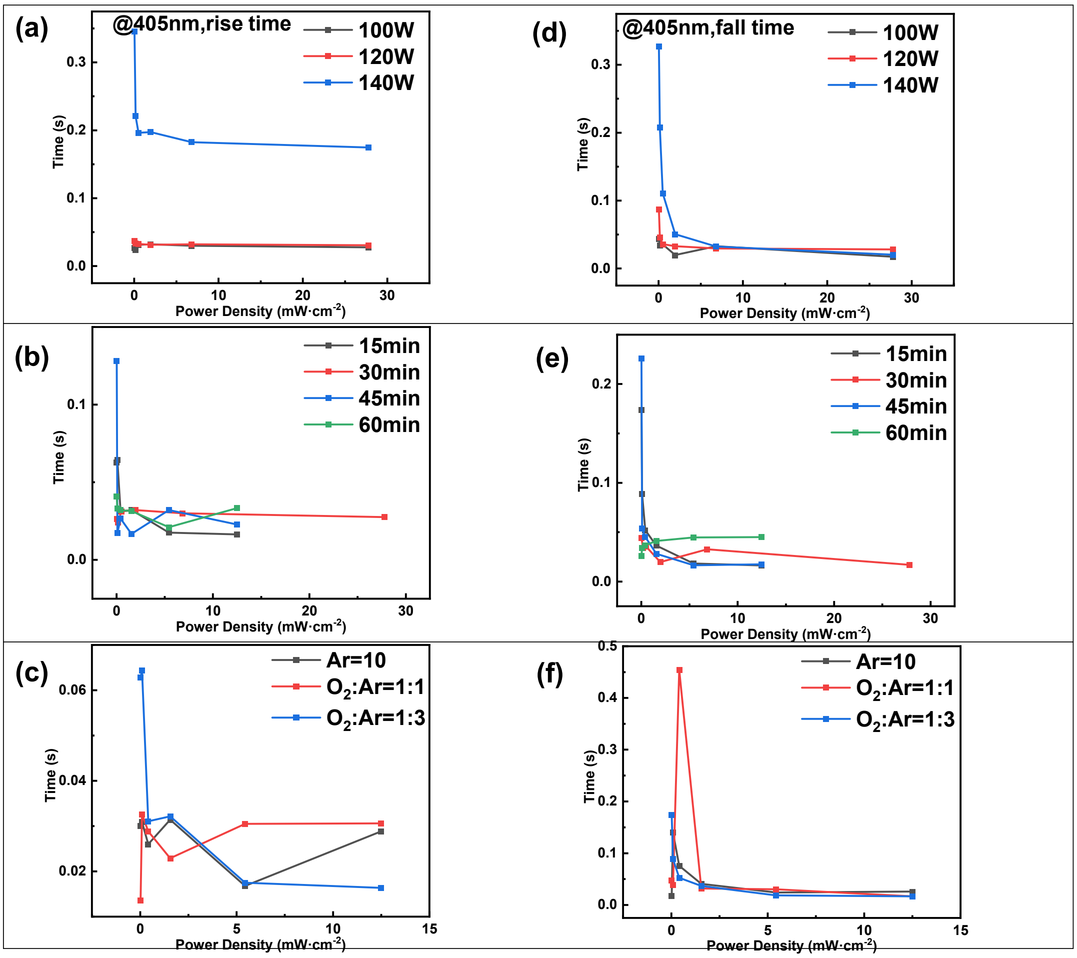

Figure S22. Device response rise time under a 405 nm illumination under different sputtering parameters: (a) sputtering time; (b) sputtering time; and (c) oxygen–argon ratio. Device response fall time under a 405 nm illumination under different sputtering parameters: (a) sputtering time; (b) sputtering time; and (c) oxygen–argon ratio.

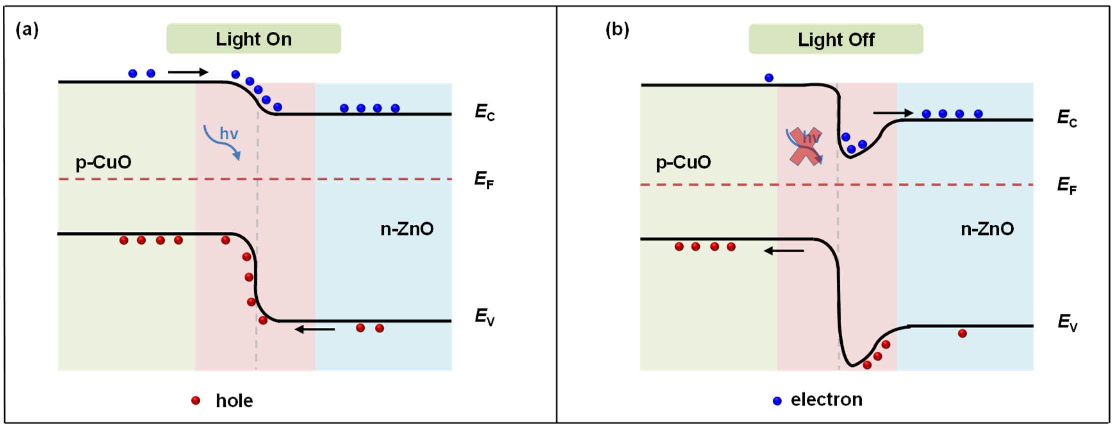

Figure S23. Schematic energy band diagram of p-CuO/n-ZnO heterojunction pyro-phototronic photodetector when light is turned on (a) and off (b).
